# CREB3 suppresses hepatocellular carcinoma progression by depressing AKT signaling through competitively binding with insulin receptor and transcriptionally activating RNA‐binding motif protein 38

**DOI:** 10.1002/mco2.633

**Published:** 2024-07-01

**Authors:** Yi He, Shenqi Han, Han Li, Yu Wu, Wenlong Jia, Zeyu Chen, Yonglong Pan, Ning Cai, Jingyuan Wen, Ganxun Li, Junnan Liang, Jianping Zhao, Qiumeng Liu, Huifang Liang, Zeyang Ding, Zhao Huang, Bixiang Zhang

**Affiliations:** ^1^ Hepatic Surgery Center Tongji Hospital, Tongji Medical College, Huazhong University of Science and Technology Wuhan China; ^2^ Department of Pediatric Surgery Tongji Hospital, Tongji Medical College, Huazhong University of Science and Technology Wuhan China; ^3^ Clinical Medical Research Center of Hepatic Surgery at Hubei Province Wuhan China; ^4^ Hubei Key Laboratory of Hepato‐Pancreatic‐Biliary Diseases, Tongji Hospital, Tongji Medical College, Huazhong University of Science and Technology Wuhan China

**Keywords:** AKT signaling, cAMP responsive element binding protein 3 (CREB3), hepatocellular carcinoma (HCC), insulin receptor (INSR), RNA‐binding motif protein 38 (RBM38)

## Abstract

cAMP responsive element binding protein 3 (CREB3), belonging to bZIP family, was reported to play multiple roles in various cancers, but its role in hepatocellular carcinoma (HCC) is still unclear. cAMP responsive element binding protein 3 like 3 (CREB3L3), another member of bZIP family, was thought to be transcription factor (TF) to regulate hepatic metabolism. Nevertheless, except for being TFs, other function of bZIP family were poorly understood. In this study, we found CREB3 inhibited growth and metastasis of HCC in vitro and in vivo. RNA sequencing indicated CREB3 regulated AKT signaling to influence HCC progression. Mass spectrometry analysis revealed CREB3 interacted with insulin receptor (INSR). Mechanistically, CREB3 suppressed AKT phosphorylation by inhibiting the interaction of INSR with insulin receptor substrate 1 (IRS1). In our study, CREB3 was firstly proved to affect activation of substrates by interacting with tyrosine kinase receptor. Besides, CREB3 could act as a TF to transactivate RNA‐binding motif protein 38 (RBM38) expression, leading to suppressed AKT phosphorylation. Rescue experiments further confirmed the independence between the two functional manners. In conclusion, CREB3 acted as a tumor suppressor in HCC, which inhibited AKT phosphorylation through independently interfering interaction of INSR with IRS1, and transcriptionally activating RBM38.

## Graphical abstract

cAMP responsive element binding protein 3 (CREB3) hinders the progression of hepatocellular carcinoma (HCC) by suppressing the phosphorylation of AKT signaling through binding competitively with insulin receptor substrate 1 (IRS1) to insulin receptor (INSR). Meanwhile, N‐terminal of CREB3 inhibits AKT activation by transcriptionally promoting RNA‐binding motif protein 38 (RBM38) expression in nucleus. Our findings provide novel insight into HCC therapy.

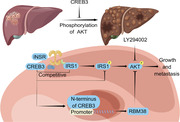

## 1 INTRODUCTION

Hepatocellular carcinoma (HCC) is one of the most common cancers worldwide. Rapid growth and metastasis of HCC lead to high mortality and recurrence.^1^ However, the mechanisms of HCC development remain to be illustrated, and treatment of HCC is still a difficult problem to be resolved.

Occurrence and development of HCC was suggested to be result of sequential accumulation of multiple genomic and epigenomic alterations.^2^, ^3^ Mutation of driver genes were classified into several major biological pathways, including AKT/mTOR, MAP kinase (MAPK), P53/‐cell cycle regulation, Wnt/β‐catenin, and so on.^4^, ^5^ HCC with activated AKT signaling displayed stronger proliferation and invasiveness.^6^ HCC therapy targeting to AKT signaling is still preliminary. It is needed to further investigate the mechanism of AKT inhibition in HCC treatment.

Insulin receptor (INSR) signaling acts as a key regulator in energy metabolism and cell growth.^7^ HCC is a primary tumor originated from liver, which highly expresses INSR.^8^ Activation of INSR signaling pathways contributes to phosphorylation of insulin receptor substrate 1 (IRS1),^9^ activation of AKT,^10^ and MAPK signaling,^11^ as well as initiation and development of HCC.^12^ Thus, it makes great significance to block INSR stimulation signals in therapy of HCC.

cAMP responsive element binding protein 3 (CREB3) is a member of transcription factor (TF) bZIP family, which includes CREB3, cAMP responsive element binding protein 3 like (CREB3L) 1, CREB3L2, CREB3L3, and CREB3L4. Effects of CREB3 family on diseases and their regulatory mechanisms vary obviously in different environment.^13^ For example, CREB3L3, also known as CREBH, was supposed to regulate hepatic metabolism through transactivating peroxisome proliferator activated receptor α and liver X receptor α. CREB3L1 was associated with extracellular matrix regulation and chondrogenesis.^14^ CREB3 was reported to have effects on progression of several cancers. Nevertheless, the role of CREB3 in cancer formation and progression is complicated. In breast cancer, CREB3 was identified as a transcriptional regulator of enhanced ADP ribosylation factor 4, COPI coat complex subunit beta 1, and USO1 vesicle transport factor, leading to promotion of endoplasmic reticulum (ER)‐Golgi trafficking and acceleration of tumor cells metastasis.^15^ However, in estrogen receptor α (ERα)‐positive breast cancer, CREB3 could bind to ERα and inhibit proliferation of breast cancer cell.^16^ In prostate cancer, CREB3 played an opposite role in different cancer subtypes. CREB3 suppressed the growth of androgen‐dependent prostate cancer by downregulation of androgen receptor (AR) transcriptional activity, but accelerated progression of androgen‐independent prostate cancer.^17^ The evidences mentioned above indicated that CREB3 had sophisticated mechanisms in control of cancer progression. However, there is still no report about function of CREB3 in HCC.

CREB3 was supposed to affect downstream signaling pathway through two methods, including interaction with receptor^16^, ^17^, ^18^ and transactivation of downstream genes.^19^ It could interact with certain receptors to influence the ligands binding, regulating related signaling pathways. Previous studies have showed that CREB3 bound to C‐C Motif Chemokine Receptor 1 and selectively enhanced chemotactic activity and Ca^2+^ mobilization of leukotactin‐1.^18^ In androgen‐dependent prostate cancer, CREB3 interacted with AR, and suppressed its transcriptional activity, leading to decreased proliferation of prostate cancer cells.^17^ Moreover, in ERα‐positive breast cancer, CREB3 could interact with ERα and suppress its binding to promoter of downstream genes in nucleus, resulting in declined growth of breast cancer.^16^ On the other hand, CREB3 could act as TF to enhance expression of downstream genes. In ER stress, CREB3 would be transferred to Golgi complex. During this progression, CREB3 is cleaved by Site‐1 protease (S1P) and Site‐2 protease (S2P).^20^ N‐terminus released from CREB3 will be imported to nucleus and transcriptionally increase downstream genes expression by binding to the promoter.^19^

In this study, we found the interaction between CREB3 and INSR, which contributed to the inhibition of INSR signaling pathway, and transactivation of RNA‐binding motif protein 38 (RBM38), resulting in dephosphorylation of AKT and suppression of HCC development. These results uncovered the role and molecular mechanism of CREB3 in HCC progression, which proposed CREB3 as a promising prognostic marker and therapeutic target for HCC.

## 2 RESULTS

### 2.1 CREB3 is downregulated in HCC and predicts better prognosis

**FIGURE 1:**
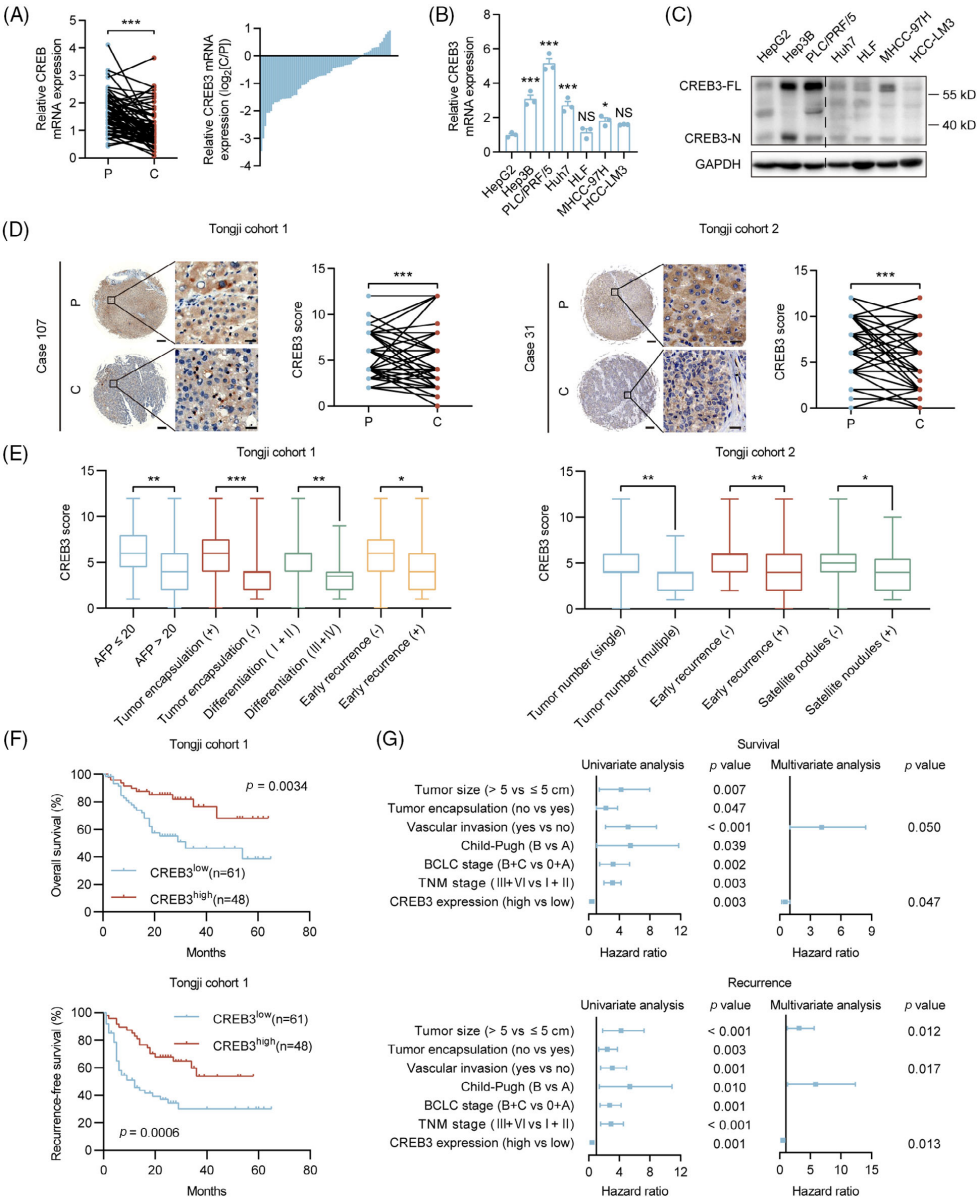
Expression of CREB3 is decreased in HCC tissues and correlated with better clinical outcome. (A) mRNA expression of CREB3 in HCC tissues and adjacent nontumor tissues. (B and C) mRNA and protein expression of CREB3 in indicated cell lines. CREB3‐FL indicated the full length of CREB3; CREB3‐N indicated N‐terminus of CREB3. GAPDH as loading control. (D) Representative images and quantification of CREB3 IHC staining in 110 paired HCC tissues and adjacent nontumor tissues. Scale bar: 200 µm, 20 µm. (E) Relative expression scores of CREB3 in HCC with or without indicated clinicopathological variables. (F) Kaplan–Meier's analysis of the correlation between CREB3 expression and the overall survival and recurrence‐free survival of patients with HCC in Tongji cohort 1. (G) Univariate and multivariate analysis of survival and recurrence displaying independent risk factors of HCC in Tongji cohort 1. Data were represented as mean ± SEM. *p < 0.05, **p < 0.01, ***p < 0.001. P, peritumor; C, cancer. NS, no significance.

To investigate the expression pattern of CREB3 in HCC, we evaluated its mRNA and protein levels in HCCs and paired peritumoral tissues. Quantitative real‐time PCR (qRT‐PCR) analysis demonstrated that the mRNA levels of CREB3 were decreased in tumor tissues compared with corresponding peritumoral tissues (Figure 1A). Western blotting and qRT‐PCR analysis showed that CREB3 was highly expressed in HCC cell lines with low invasiveness, including Hep3B, PLC/PRF/5, Huh7, and was relatively less expressed in cell lines with high invasiveness including HLF, MHCC‐97H (97H), and HCC‐LM3 (LM3)^21^, ^22^ (Figure 1B,C). Immunohistochemistry (IHC) analysis for two HCC tissue microarrays (TMAs) (Tongji cohort 1 and Tongji cohort 2) showed that the in situ protein level of CREB3 was lower in HCCs compared with peritumoral tissues (Figure 1D). In addition, HCC tissues of patients with higher alpha fetoprotein (AFP) level (>20 µg/L), incomplete tumor encapsulation, poorer differentiation grade (III and IV), earlier recurrence (<2 years), multiple tumor numbers, and satellite nodules showed lower CREB3 staining intensity (Figure 1E). Chi‐square analysis exhibited that lower expression of CREB3 was associated with larger tumor size, vascular invasion, and advanced Barcelona Clinic Liver Cancer stage in Tongji cohort 1 (Tables S1 and S2). Kaplan–Meier analysis showed that high expression of CREB3 predicted longer overall survival (OS) and recurrence‐free survival (RFS) in Tongji cohort 1 (Figure 1F). For further exploration, we performed univariate and multivariate cox regression analysis of factors associated with survival and recurrence of patients with HCC in cohort 1 (Table S3). Multivariate analysis showed lower expression of CREB3 was an independent risk factor for shorter OS and RFS (Figure 1G). Taken together, we found that CREB3 was downregulated in HCC and low expression of CREB3 in HCC predicted poorer prognosis.

### 2.2 CREB3 inhibits proliferation and mobility of HCC cell lines

To identify the role of CREB3 in HCC, stable cell lines with CREB3 knockdown or overexpression were constructed. Hep3B in which CREB3 was highly expressed was chosen to establish HCC cells with CREB3 stably knocked down, while LM3 in which CREB3 was lowly expressed was used for construction of CREB3 stably overexpressed cell line. HLF that showed moderate CREB3 expression was used for either knockdown or overexpression. qRT‐PCR and western blotting analysis were performed to verify the efficiency of CREB3 knockdown and overexpression (Figure S1A–D).

**FIGURE 2:**
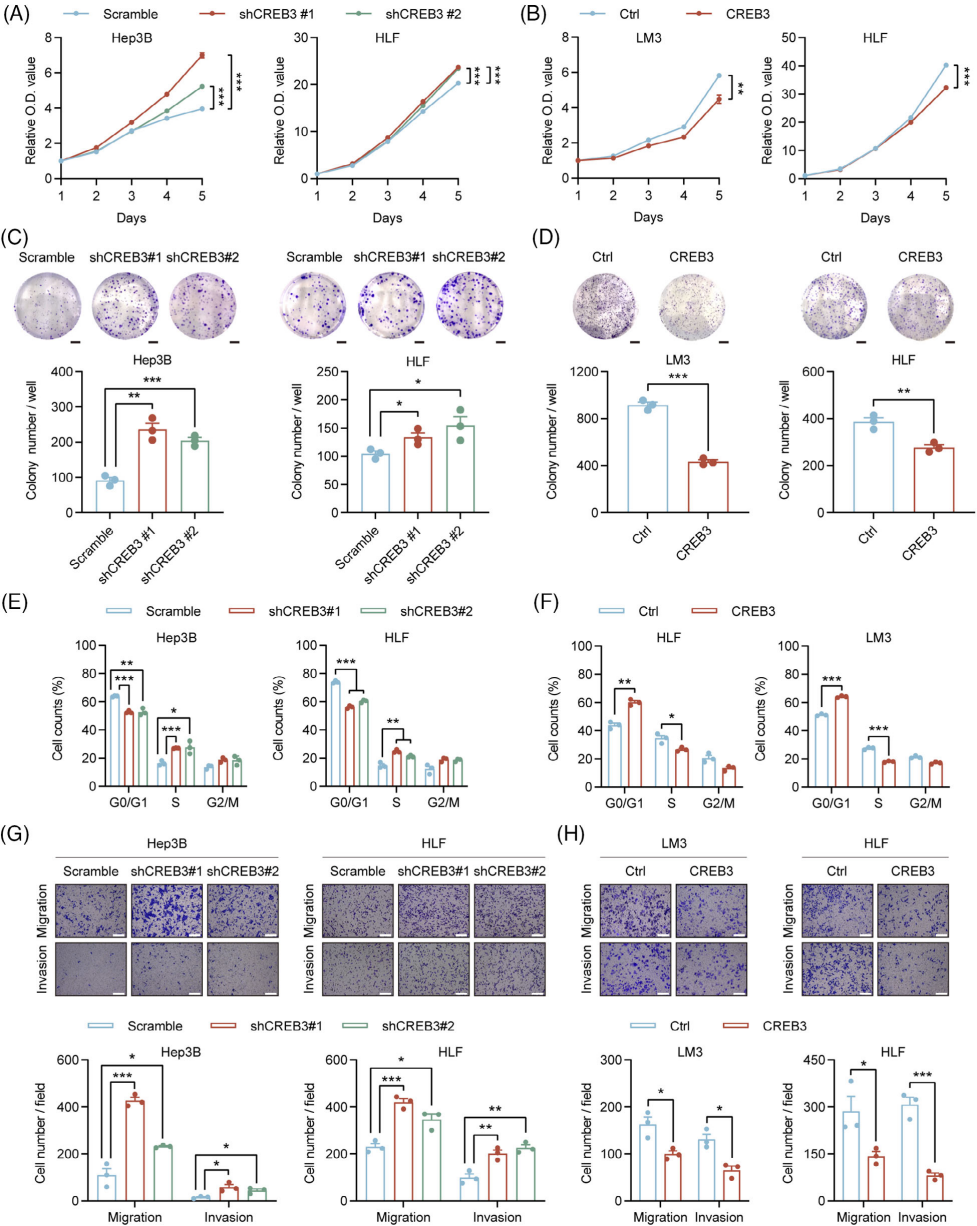
CREB3 suppresses proliferation and mobility of HCC cells. (A and B) CCK‐8 assays in Hep3B, HLF, and LM3 cells with CREB3 knockdown or overexpression (*n* = 4). (C and D) Representative images and quantification of colony formation assays in indicated cells are shown (*n* = 3). (E and F) Flow cytometry analysis of cell cycle in indicated cells with CREB3 knockdown or overexpression (*n* = 3). (G and H) Representative images of migration and invasion assays after CREB3 stably knocked down or overexpressed in Hep3B, HLF, and LM3 cells (upper panel). Scale bar: 25 µm. Quantification of indicated cells migrated or invaded (lower panel, *n* = 3). Data were represented as mean ± SEM. *p < 0. 05, **p < 0.01, ***p < 0.001. Ctrl, control; sh, short hairpin.

Cell Counting Kit‐8 (CCK‐8) assays demonstrated that HCC cells with relatively lower expression of CREB3 exhibited stronger proliferation abilities, and vice versa (Figure 2A,B). Colony formation assays suggested that Hep3B and HLF cells with CREB3 knockdown displayed more and larger cell clones, and consistent results were obtained in LM3 and HLF cells with CREB3 overexpression (Figure 2C,D). In addition, Gene Set Enrichment Analysis for liver hepatocellular carcinoma (LIHC) cohort in The Cancer Genome Atlas (TCGA) database showed that high expression of CREB3 was inversely correlated with cell cycle process (Figure S2A). To further explore the influence of CREB3 on cell cycle transition, we detected cyclin‐dependent kinase 4 and CyclinD1 (G1 to S phase transition promoter) expression in HCC cells. The results indicated that knockdown of CREB3 enhanced their expression, and vice versa (Figure S2B). Consistently, flow cytometry analysis showed that knockdown of CREB3 increased proportion of S phase and decreased proportion of G1 phase in HCC cells (Figures 2E and S2C). On the contrary, overexpression of CREB3 decreased proportion of S phase and increased proportion of G1 phase (Figures 2F and S2C). Additionally, flow cytometry was performed for apoptosis analysis. However, the results did not support the inhibitory effects of CREB3 on HCC (Figure S2D,E). Besides, transwell assays showed that HCC cells with lower CREB3 level exhibited stronger migratory and invasive abilities, and vice versa for HCC cells with higher CREB3 expression (Figure 2G,H). Epithelial–mesenchymal transition (EMT)‐related markers were detected by western blotting, indicating that CREB3 decreased the expression of N‐Cadherin, Slug and Snail, increased expression of E‐Cadherin, and depressed process of EMT (Figure S2F).

### 2.3 CREB3 suppresses growth and metastasis of HCC

**FIGURE 3:**
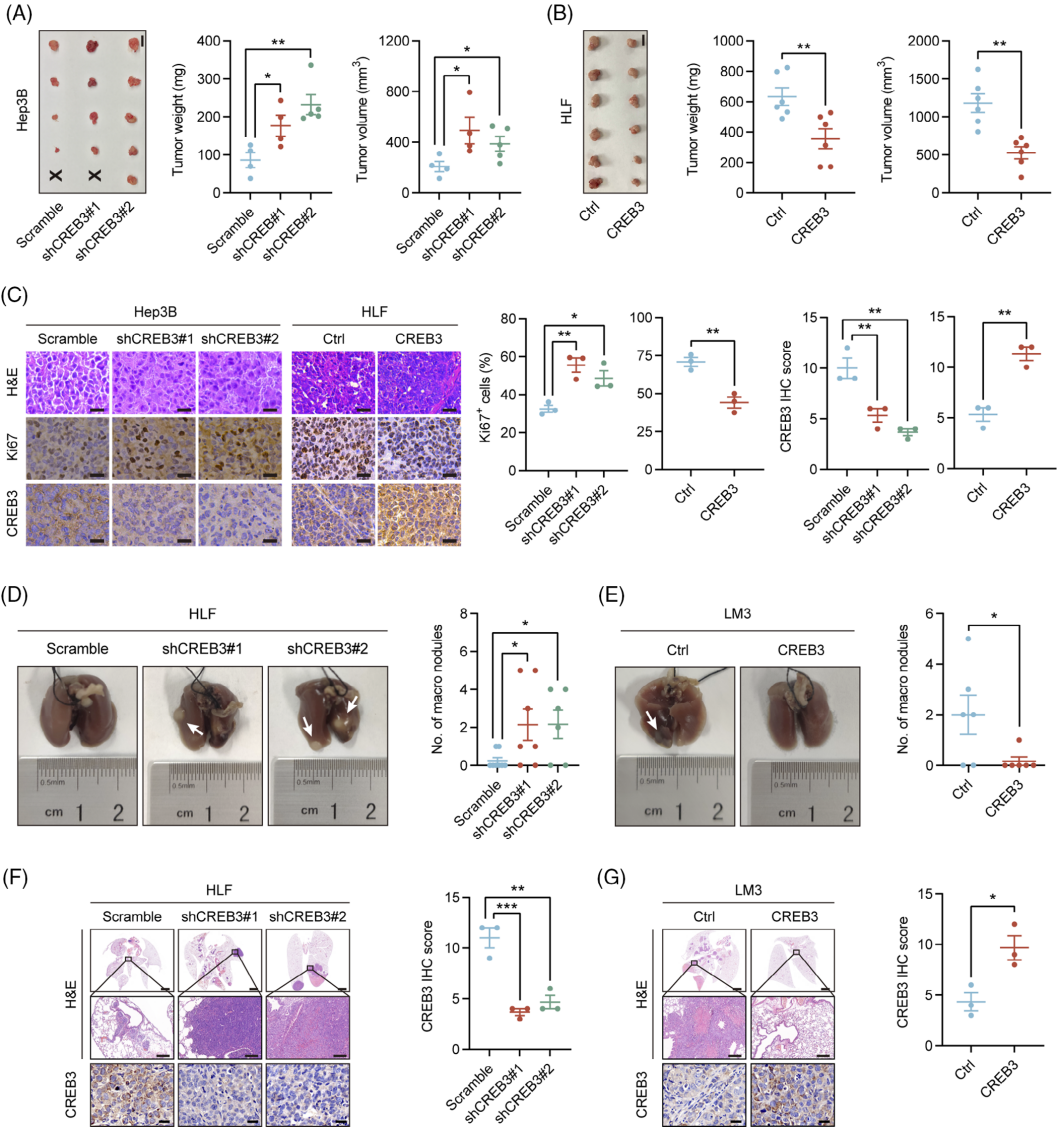
CREB3 inhibits tumorigenesis and lung metastasis in vivo. (A and B) Image of subcutaneous tumors from Hep3B CREB3 stably knocked down (*n* = 4 in Scramble or shCREB3#1, *n* = 5 in shCREB3#2) or HLF CREB3 overexpressed (*n* = 6). Statistical comparison of tumors weight and volume of indicated groups were performed. (C) Representative images of H&E, CREB3, and Ki67 staining of subcutaneous tumors in the indicated groups. Scale bar: 25 µm. Quantification of CREB3 IHC scores and Ki67^+^ HCC cell percentages (*n* = 3). (D and E) Representative images and quantification of lung metastases in nude mice with CREB3 knockdown (*n* = 8 in Scramble, *n* = 7 in shCREB3#1, *n* = 6 in shCREB3#2) or overexpression (*n* = 6). (F and G) Representative images of H&E and CREB3 staining in lung metastatic nodules of indicated groups. Scale bar: 2 mm (upper), 200 µm (middle), 20 µm (lower). Quantification of CREB3 IHC scores (*n* = 3). Data were represented as mean ± SEM. *p < 0.05, **p < 0.01, ***p < 0.001. Ctrl, control; sh, short hairpin.

To explore whether CREB3 had effects on growth of HCC in vivo, nude mice were subcutaneously injected with HCC cells with CREB3 stably knocked down (Hep3B/shCREB3) or overexpressed (HLF/CREB3). Tumor weight and volume were significantly higher in Hep3B/shCREB3 group compared with control group (Hep3B/scramble) (Figure 3A). In contrast, overexpression of CREB3 remarkably decreased the tumor weight and volume of HLF cells (Figure 3B). Meanwhile, Ki67 staining demonstrated that HCC cells with lower CREB3 expression (Hep3B/shCREB3 and HLF/Ctrl) displayed higher proliferative activities (Figure 3C).

For the reason that HLF and LM3 cell lines were reported to have better metastatic ability,^21^, ^22^ we chosen HLF with CREB3 stably knocked down and LM3 with CREB3 overexpressed to perform lung metastasis assay. HLF or LM3 was injected into tail veins of nude mice. After 8 weeks, all mice were sacrificed. More metastatic nodules were observed in mice injected with HCC cells of lower CREB3 expression (HLF/shCREB3 and LM3/Ctrl) (Figure 3D–G).

### 2.4 CREB3 inhibits HCC progression through AKT signaling pathway

**FIGURE 4:**
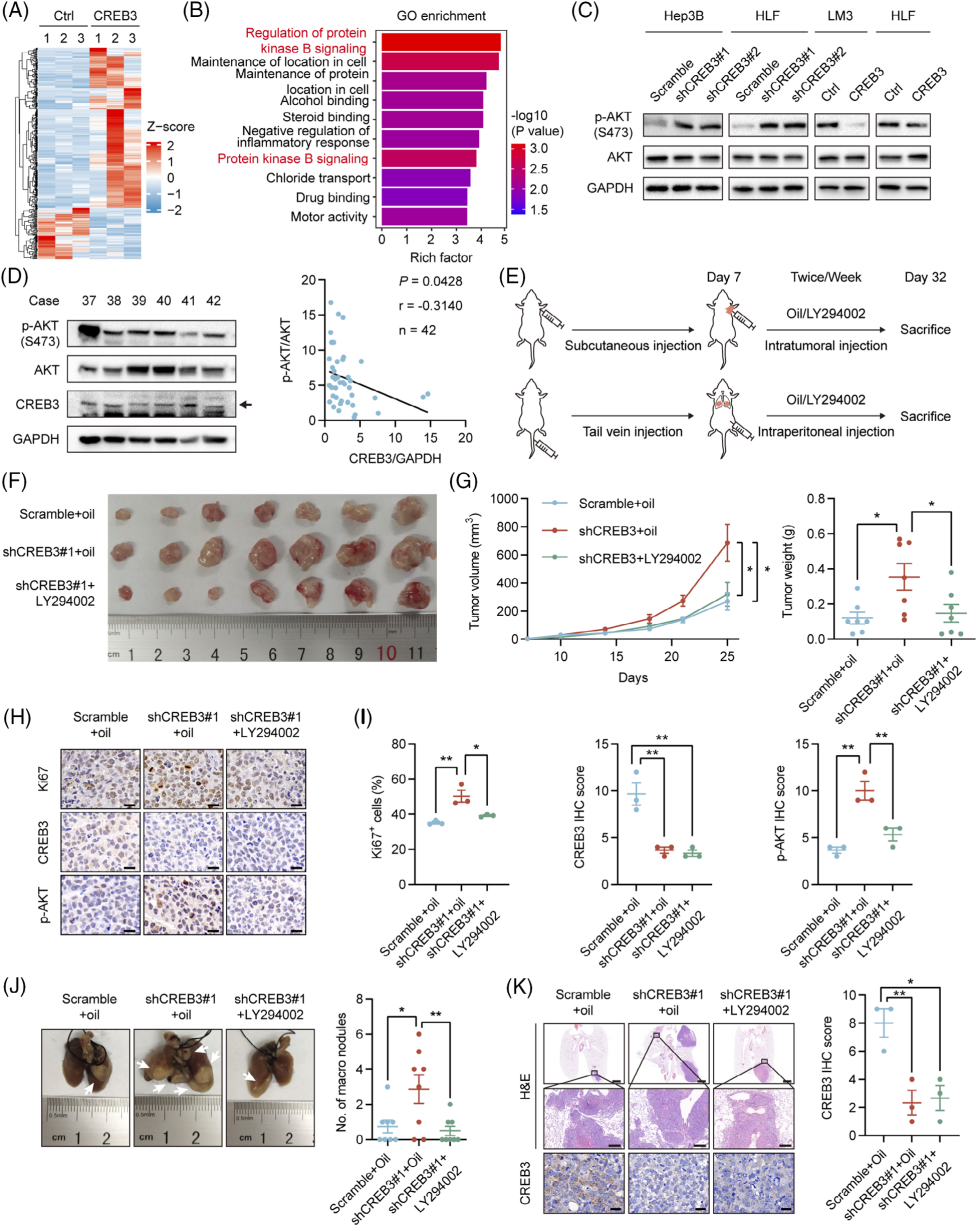
CREB3 suppresses growth and metastasis of HCC through AKT signaling pathway. (A) Heat map displaying the DEGs between Ctrl and CREB3 overexpression of HLF cells. (B) GO analysis of DEGs were shown. Protein kinase B signaling related terms are marked in red. (C) Western blotting analysis of indicated protein in CREB3 stably knocked down or overexpressed HCC cells. (D) Representative western blotting images of indicated protein in clinical HCC samples. Scatterplots showing the robust correlation of CREB3 and p‐AKT (Ser^473^)/AKT protein expression. Expression of CREB3, p‐AKT (Ser^473^), and AKT were normalized to GAPDH. (E) HLF cells with or without knockdown of CREB3 were injected subcutaneously (*n* = 7) or intravenously (*n* = 8) into BALB/c nude mice. Seven days later, mice were injected intratumorally or intraperitoneally with oil or LY294002 twice a week. (F) Image of subcutaneous tumors from HLF cells with or without CREB3 stably knocked down injected with or without LY294002. (G) Statistical comparison of tumors weight and volume of indicated groups were performed. (H) Representative images of Ki67, CREB3, and p‐AKT (Ser^473^) staining of subcutaneous tumors in the indicated groups. Scale bar: 20 µm. (I) Quantification of Ki67^+^ HCC cell percentages, CREB3, and p‐AKT (Ser^473^) IHC scores in indicated groups (*n* = 3). (J) Representative images and statistical comparison of lung metastatic nodules in CREB3 control or knocked down nude mice with or without LY294002 injection. (K) Representative images of H&E, CREB3 staining in lung metastatic nodules of the indicated groups. Scale bar: 2 mm (upper), 200 µm (middle), 20 µm (lower). Quantification of CREB3 IHC scores was shown (*n* = 3). Data were represented as mean ± SEM. *p < 0.05, **p < 0.01. sh, short hairpin.

To explore how CREB3 inhibited progression of HCC, mRNA of HLF/Ctrl and HLF/CREB3 was extracted and subjected to mRNA sequencing. A total of 263 differentially expressed genes (DEGs) (fold change > 1.5, *p* value < 0.05) were identified (Figure 4A) and were further analyzed by Gene Ontology (GO) enrichment analysis (Table S4), as well as Kyoto Encyclopedia of Genes and Genomes (KEGG) pathway enrichment analysis (Table S5). We noticed protein kinase B signaling, also known as AKT signaling, was enriched as the top term with highest rich factor in GO enrichment (Figure 4B). It was known that AKT signaling was reported to play a crucial role in HCC progression.^23^, ^24^ To identify the relationship between CREB3 and AKT signaling, western blotting analysis was conducted to detect the phosphorylation of AKT in CREB3 knocked down or overexpressed HCC cells. The result indicated that knockdown of CREB3 increased the phosphorylation of AKT, and vice versa (Figure 4C). Additionally, we detected CREB3 expression and AKT phosphorylation in clinical HCC samples (Figure S3A). The result indicated negative correlation between CREB3 and AKT phosphorylation (Figure 4D). Therefore, we focused on AKT signaling for further study. First of all, inhibitory effects of LY294002 on phosphorylation of AKT was verified in HLF cells (Figure S3B). To further confirm CREB3 suppressed tumor growth and metastasis through AKT signaling, LY294002 was used to inhibit AKT activity in vivo for rescue animal experiments. We injected LY294002 into subcutaneous tumors for transplantation tumor experiment or peritoneal cavity for lung metastasis experiment twice a week. After 25 days, all mice were sacrificed, and tumors were taken out for study (Figure 4E). Mice bearing HLF/shCREB3 cells developed larger and heavier tumors than control group (HLF/Scramble), while LY294002 treatment attenuated these effects (Figure 4F,G). IHC staining suggested that the increasement of Ki67 and phosphorylated AKT, which were induced by CREB3 knockdown, were inversed by LY294002 treatment (Figure 4H,I). In addition, mice that were injected with HLF/shCREB3 cells by tail‐vein developed more lung metastatic nodules than control group (HLF/Scramble), while administrating with LY294002 resulted in decreased lung metastases compared with mice injected with HLF/shCREB3 cells (Figure 4J,K). Therefore, we demonstrated that CREB3 suppressed HCC progression through AKT signaling pathway.

### 2.5 CREB3 decreases phosphorylation of AKT by competitively binding to INSR

**FIGURE 5:**
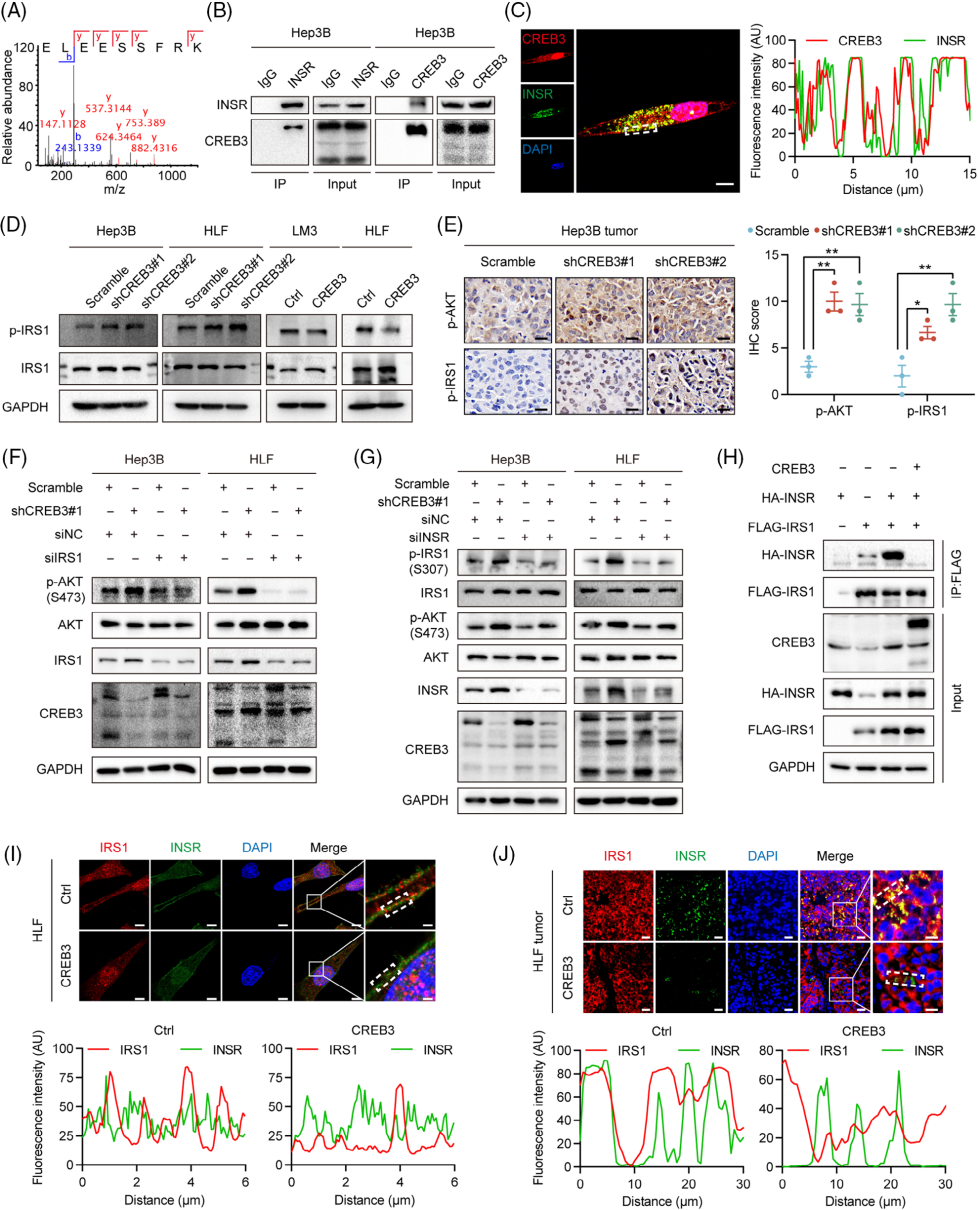
CREB3 decreases AKT phosphorylation through competitively binding to INSR. (A) The peptide fragment of INSR as determined by mass spectrometry. (B) Co‐IP analysis of the binding between endogenous INSR and CREB3 in Hep3B cells. (C) IF staining and line scans analysis of signal intensity using CREB3 antibody (red) and INSR antibody (green) in Hep3B cells. Nuclei were counterstained with DAPI (blue). Scale bar: 10 µm. (D) Western blotting analysis of indicated protein in CREB3 stably knocked down or overexpressed HCC cells. (E) Representative images and quantification of p‐AKT (Ser^473^) and p‐IRS1 (Ser^307^) staining of subcutaneous tumors in the indicated groups (*n* = 3). Scale bar: 20 µm. (F) Western blotting analysis of indicated protein in CREB3 stably knocked down or negative control Hep3B and HLF cells with IRS1 siRNA or negative control treatment for 48 h. (G) Western blotting analysis of indicated protein in CREB3 stably knocked down or negative control Hep3B and HLF cells with INSR siRNA or negative control treatment for 48 h. (H) Co‐IP analysis of the binding between exogenous HA‐INSR and FLAG‐IRS1 in cotransfected 293T cells with CREB3 or empty vector transfection. (I and J) Representative confocal images and line scans analysis of IRS1 (red) and INSR (green) staining in HLF cells (I) and HLF subcutaneous tumors (J) with CREB3 overexpression or control. Nuclei were counterstained with DAPI (blue). Scale bar: 10 µm, 2 µm in (J); 20 µm, 10 µm in (K). GAPDH as loading control in (D, F, G, and H). *p < 0.05, **p < 0.01. sh, short hairpin.

Based on previous study, CREB3 was supposed to affect downstream signaling pathway through two methods, including interaction with receptor^16^, ^17^, ^18^ and transactivation of downstream genes.^19^ To find out how CREB3 regulated AKT signaling, we explored function of CREB3 in aspects of protein interaction and transcriptional activity. To uncover potential interacting proteins, the products of co‐immunoprecipitation (co‐IP) assay using anti CREB3 antibody in CREB3 overexpressed HEK293T (293T) cells were subjected to mass spectrometry (MS). To screening potential interacting proteins, we excluded proteins overlapping in IgG group and filtered out proteins with less than 10 unique peptides detected and obtained seven candidate interacting proteins (Table S6). Next, we removed nonspecific proteins including keratins, albumin, and ribonucleoproteins, from the candidate proteins, and then INSR and host cell factor 1 (HCFC1) stood out. In addition, previous studies have already reported the interaction of CREB3 and HCFC1.^25^, ^26^ Thus, we chose INSR for further investigation (Figure 5A). co‐IP assay was performed to confirm the interaction between CREB3 and INSR. FLAG‐INSR and CREB3 overexpression plasmids were transiently transfected into 293T cells, and co‐IP assay indicated that CREB3 bound to INSR in 293T cells (Figure S4A). Endogenous interaction of CREB3 and INSR was further validated in Hep3B cells using anti‐INSR and anti‐CREB3 antibody (Figure 5B). Immunofluorescence (IF) staining also showed that CREB3 colocalized with INSR in Hep3B cells (Figure 5C).

As is known, activated INSR can recruit and phosphorylate insulin receptor substrate 1 (IRS1) to initiate INSR signaling pathway.^9^, ^10^ Phosphorylation of IRS1, which is a main protein in INSR signaling pathway,^9^ was detected by western blotting in HCC cells. The result indicated that CREB3 inhibited IRS1 phosphorylation in HCC cell lines (Figure 5D). IHC analysis of subcutaneous tumor and lung metastasis tissues also revealed that CREB3 expression was inversely correlated with phosphorylation of IRS1 and AKT (Figures 5E and S4B,C). In addition, silencing IRS1 prevented the upregulation of AKT phosphorylation induced by knockdown of CREB3 in Hep3B and HLF cells (Figure 5F). Downregulation of INSR attenuated the increased phosphorylation of IRS1 induced by CREB3 knockdown (Figure 5G). These results suggested that CREB3 suppressed phosphorylation of AKT through INSR and IRS1. IRS1 was previously reported to be activated by INSR.^9^ Thus, the role of CREB3 interacting with INSR and inhibiting IRS1 phosphorylation implied us to explore whether CREB3 interfered the binding between INSR and IRS1. Then, HA‐INSR and FLAG‐IRS1 plasmids were cotransfected into 293T cells. We found that overexpression of CREB3 did impair the binding between INSR and IRS1 (Figure 5H). Also, IF staining revealed that CREB3 overexpression decreased IRS1 colocalization with INSR in HLF cells and LM3 cells (Figures 5I and S4D), as well as in HLF subcutaneous tumor tissues (Figure 5J). To sum up, these results indicated that CREB3 competitively bound to INSR and reduced phosphorylation of IRS1, which led to downregulated phosphorylation of AKT.

### 2.6 CREB3 transactivates RBM38 expression to decrease the phosphorylation of AKT in HCC

**FIGURE 6:**
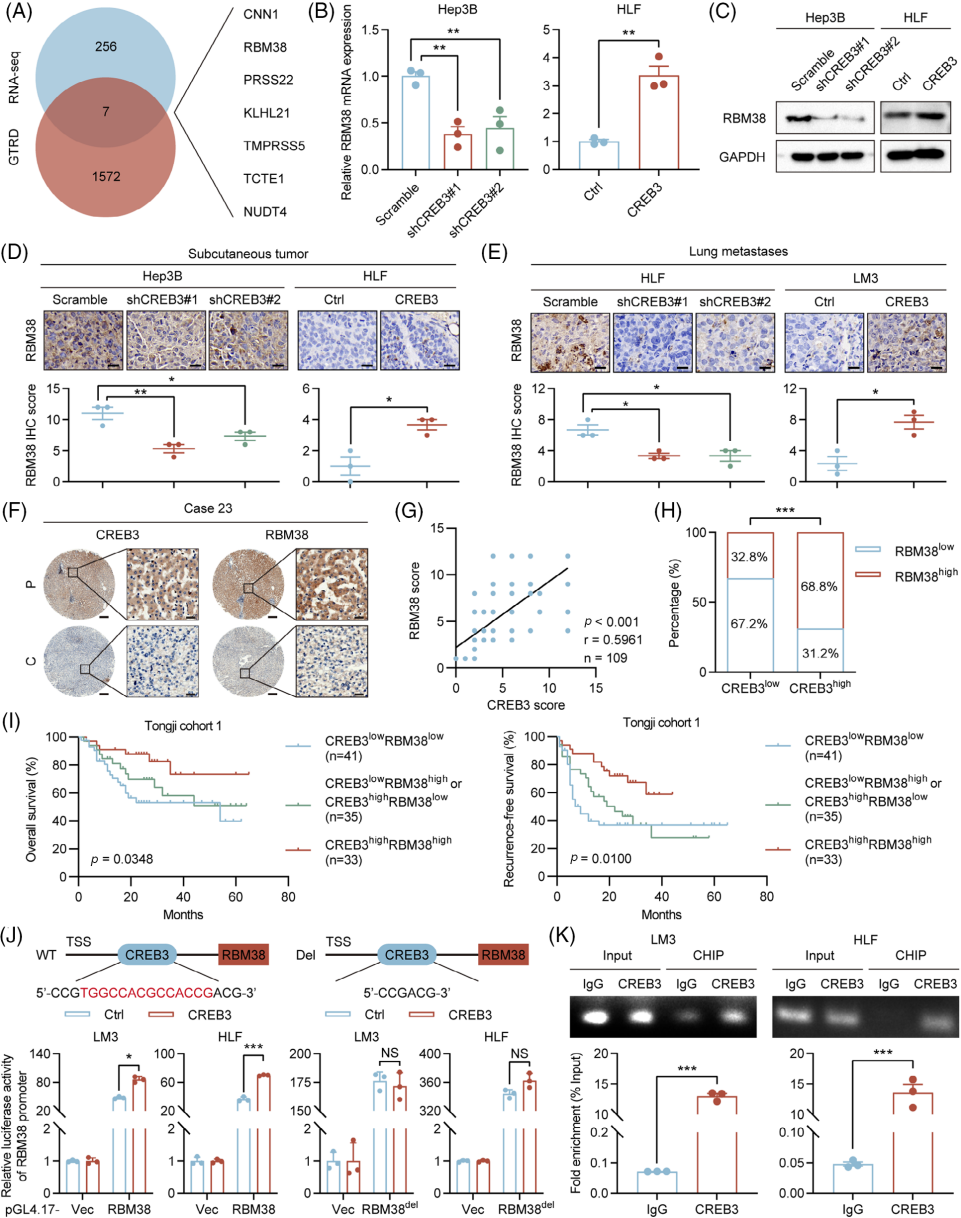
CREB3 transactivates RBM38 expression to decrease the phosphorylation of AKT. (A) Overlap of the predicted genes transactivated by CREB3 from GTRD and DEGs from mRNA sequencing. (B and C) qRT‐PCR and western blotting analysis of RBM38 mRNA and protein levels in Hep3B or HLF cells with CREB3 stably knocked down or overexpressed (*n* = 3). Data were shown as the fold change to their respective control cells in (B) and were normalized to GAPDH in (C). (D and E) Representative images and quantification of RBM38 staining of subcutaneous tumors and lung metastases in the indicated groups (*n* = 3). Scale bar: 20 µm. (F) Representative IHC staining images of CREB3 and RBM38 in paired HCC samples (*n* = 110) (Tongji cohort 1). Scale bar: 200 µm, 25 µm. (G) Scatterplots showing the robust correlation of CREB3 and RBM38 IHC scores. Statistical analysis was performed using Pearson's correlation. (H) Respective percentages and chi‐square test of RBM38 low or high expression in CREB3 low or high expression group. (I) Kaplan–Meier analysis of the overall and recurrence‐free survival of HCC patients stratified by CREB3 and RBM38 expression levels in Tongji cohort 1. (J) Relative luciferase activities of Control or CREB3 overexpressed LM3 or HLF cells transfected with pGL4.17‐Vec, pGL4.17‐RBM38, or pGL4.17‐RBM38^del^. The sequence of CREB3 binding site on RBM38 promoter is marked in red. Renilla luciferase was used as loading control. (K) Endogenous ChIP experiment of CREB3 binding to RBM38 promoter in LM3 or HLF cells. qRT‐PCR detection and agarose gel electrophoresis of PCR fragments after ChIP. Data were represented as mean ± SEM. *p < 0.05, **p < 0.01, ***p < 0.001. TSS, transcription start site; Ctrl, control; sh, short hairpin; WT, wild type; Del, delete; NS, no significance.

To explore the function of CREB3 in aspect of transcriptional activity, we tried to identify whether CREB3 could enter nucleus and function as a transcriptional factor (TF) in HCC. As is known, CREB3 could be cleaved by S1P and S2P.^20^ Thus, Brefeldin A (BFA) was used to induce cleavage of CREB3. Western blotting showed N‐terminus of CREB3, which had transcriptional activity, was increased with stimulation of BFA in Hep3B and LM3 (Figure S5A). IF staining suggested that N‐terminus of CREB3 accumulated in nucleus with treatment of BFA (Figure S5B). Gene Transcription Regulation Database (GTRD),^27^ which was based on chromatin immunoprecipitation (ChIP)‐seq data, was used to predict target genes whose promoter might be bound by CREB3. The intersection of DEGs in CREB3 mRNA sequencing and genes predicted by GTRD database was figured out, including RBM38, kelch like family member 21, serine protease 22, calponin 1, t‐complex‐associated‐testis‐expressed 1, transmembrane serine protease 5, and nudix hydrolase 4 (Figure 6A). To screen target genes of CREB3, we performed qRT‐PCR to verify each of candidate genes (Figures 6B and S5C). Expression of RBM38 showed the most remarkable variation according to knockdown or overexpression of CREB3. So RBM38 was chosen for further study. qRT‐PCR and western blotting analysis suggested that both of mRNA and protein expression of RBM38 were reduced in CREB3 knockdown HCC cells but were increased in CREB3 overexpressed cells, which implied CREB3 could transcriptionally regulate RBM38 (Figures 6B,C and S5D). In addition, effects of CREB3 on RBM38 was verified by IHC staining in mice tumor slices with CREB3 overexpression or knockdown (Figure 6D,E). Moreover, IHC staining of RBM38 in TMAs of Tongji cohort 1 showed the expression of CREB3 was positively correlated with RBM38 level (*p* < 0.001, *r* = 0.5961) (Figure 6F–H). Similar results were obtained in TCGA‐LIHC and Gene Expression Omnibus cohorts (Figure S6). Prognosis analysis indicated that patients with both high expression of CREB3 and RBM38 (cutoff value = 4) displayed the best OS and RFS, and patients with both low expression of CREB3 and RBM38 showed the worse outcomes (Figure 6I). The evidences mentioned above illustrated that CREB3 positively regulated mRNA and protein level of RBM38, which strongly benefited the prognosis of HCC patients.

In addition, dual luciferase reporter assay was performed to investigate transcriptional activation activity of CREB3 on RBM38. JASPAR database^28^ was used to predict the binding site of CREB3 on RBM38 promoter. Luciferase vector plasmid with RBM38 promoter was constructed and transfected into HCC cells. Overexpression of CREB3 promoted luciferase activity of RBM38 promoter in LM3 and HLF cells, which could be rescued by deletion of CREB3 binding site on RBM38 promoter (RBM38^del^) (Figure 6J). In 293T and HCC cells, ChIP assay indicated that CREB3 could bind to RBM38 promoter at predicted binding site, which was detected by specific primers (Figures S7A and 6K). Moreover, rescue experiment indicated that overexpression of RBM38 partially rescued enhanced phosphorylation of AKT induced by CREB3 knockdown in Hep3B and HLF (Figure S7B). Therefore, CREB3 could activate RBM38 expression in transcriptional level to decrease the phosphorylation of AKT in HCC.

### 2.7 Knockdown of INSR or overexpression of RBM38 independently attenuates tumor‐promoting action caused by CREB3 knockdown

**FIGURE 7:**
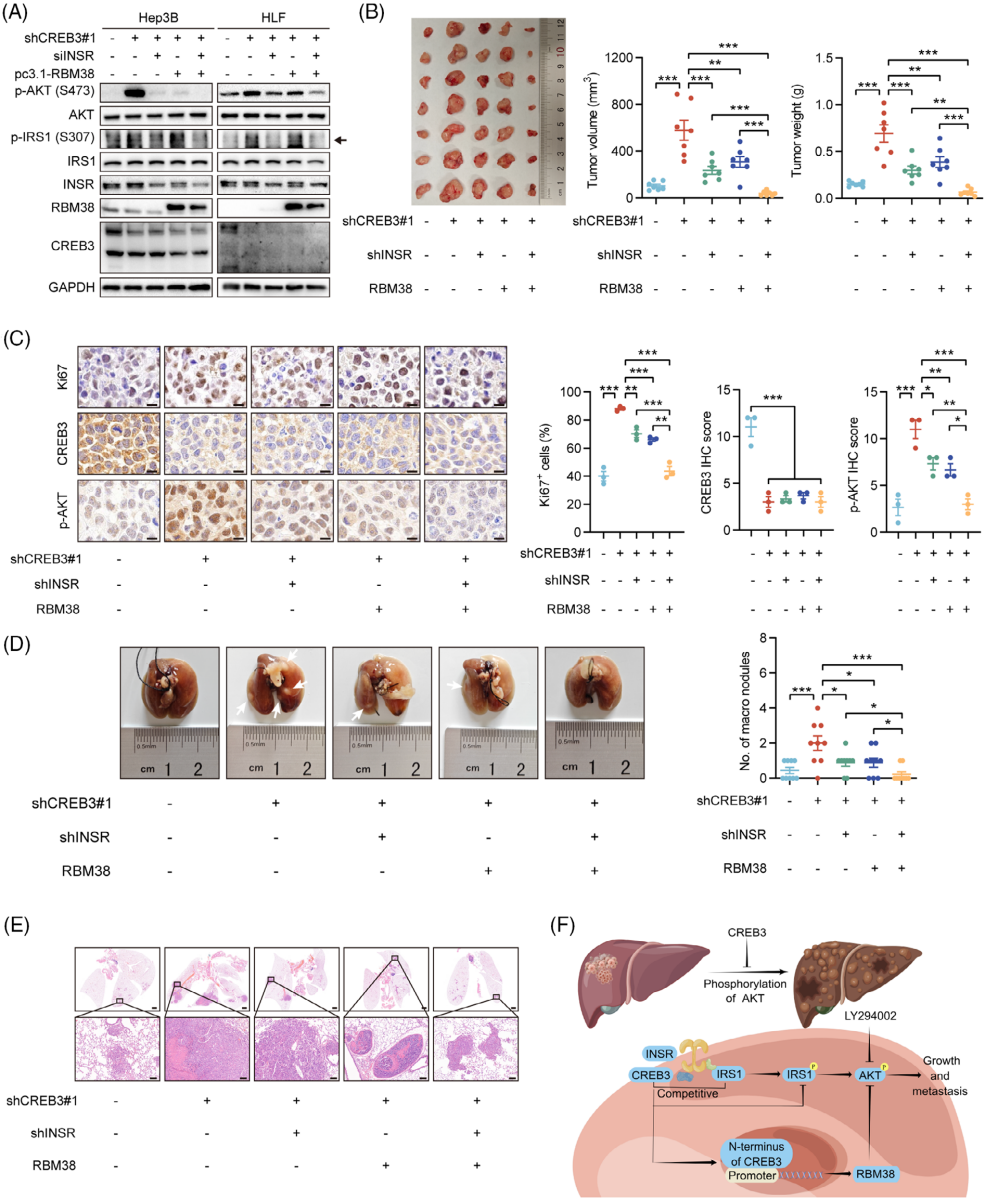
Knockdown of INSR or overexpression of RBM38 independently decreases growth and metastasis of HCC accelerated by reduced CREB3 expression. (A) Western blotting analysis of p‐AKT (Ser^473^), total AKT, p‐IRS1 (Ser^307^), total IRS1, INSR, RBM38, CREB3, and GAPDH in indicated group. GAPDH as loading control. (B) Image of subcutaneous tumors in HLF/scramble or HLF/shCREB3#1 nude mice with or without knockdown of INSR or/and overexpression of RBM38. Statistical comparison of tumor weight and volume of indicated groups were performed. (C) Representative images of Ki67, CREB3, and p‐AKT (Ser^473^) staining of subcutaneous tumors in the indicated groups. Scale bar: 10 µm. Quantification of Ki67^+^ HCC cell percentages, CREB3, and p‐AKT (Ser^473^) IHC scores in indicated groups (*n* = 3). (D) Representative images and statistical comparison of lung metastatic nodules in scramble or CREB3 knocked down nude mice with or without knockdown of INSR or/and overexpression of RBM38. (E) Representative images of H&E in lung metastatic nodules of the indicated groups. Scale bar: 1 mm (upper), 100 µm (lower). (F) Schematic model depicting the antitumor effect of CREB3 in HCC by inhibiting the phosphorylation of AKT. Data were represented as mean ± SEM. *p < 0.05, **p < 0.01, ***p < 0.001. sh, short hairpin.

To address the relationship between INSR and RBM38, rescue experiments were carried out in vitro and in vivo. siRNAs targeting to INSR or pc3.1‐RBM38 plasmids were transfected into Hep3B and HLF cells with CREB3 knocked down. Western blotting analysis showed that silencing of INSR markedly attenuated increasement of phosphorylated IRS1 induced by CREB3 knockdown, and partly suppressed AKT phosphorylation. Overexpression of RBM38 also partly reduced activity of AKT signaling. But, no crosstalk of INSR signaling and RBM38 was found so that we thought INSR signaling and RBM38, both of which were regulated by CREB3, independently affected AKT signaling (Figure 7A). Then, animal rescue experiments were conducted using HLF/shCREB3 with INSR knockdown or/and RBM38 overexpression (Figure S8A–C). Tumorigenicity assays in nude mice indicated that tumor growth induced by CREB3 knockdown could be partly rescued by decreased INSR expression or enhanced RBM38 expression. shCREB3 + shINSR + RBM38 group showed strongest inhibitory effects on tumor growth. Besides, there was no difference between shCREB3 + shINSR and shCREB3 + RBM38 group, indicating INSR and RBM38 independently modulated HCC progression (Figure 7B). IHC analysis suggested that Ki67 and phosphorylated AKT were increased by CREB3 knockdown, which were rescued by INSR knockdown or RBM38 overexpression independently (Figure 7C). Lung metastasis assay also identified the parallel roles of INSR and RBM38 in the regulation mechanism of CREB3 (Figure 7D,E). In conclusion, our study demonstrated that CREB3 suppressed the phosphorylation of AKT by competitively binding with INSR and promoting RBM38 transcription independently, thereby inhibiting HCC growth and metastasis (Figure 7F).

## 3 DISCUSSION

In the present study, we revealed CREB3 as a novel tumor suppressor in HCC. It was significantly decreased in HCC tissues from patients in both mRNA and protein levels. Patients with high expression of CREB3 were suggested to have better prognosis. CREB3 showed strongly inhibitive effects on growth and metastasis of HCC in vitro and in vivo, due to decreasing the phosphorylation of AKT by competitively binding to INSR and transcriptionally activating RBM38 independently. These results extend the understanding of HCC growth and metastasis mechanisms and may provide new therapy strategies for HCC patients.

HCC was classified into two major classes, including proliferation class and nonproliferation class. Progenitor subclass, which belongs to proliferation class, is less differentiated and displays overexpression of progenitor markers, such as insulin like growth factor 2 (IGF2), epithelial cell adhesion molecule, AFP, or keratin 19.^29^, ^30^, ^31^ AKT signaling is specifically activated in progenitor subclass and strongly enhances HCC progression.^6^, ^29^ Therapy targeting to AKT signaling inhibits growth, metastasis, and survival of tumor cells, as well as modulates tumor microenvironment.^32^ There is no doubt that AKT signaling is extremely important in HCC development. In our study, we summarized that CREB3 affected signaling pathway through two methods, including interaction with receptor and transactivation of downstream genes, which had independent effect on progression of tumor. We explored the possibility of the two methods in HCC and identified that CREB3 suppressed phosphorylation of AKT through both interaction with INSR and transactivation of RBM38 independently. This view was further identified by animal experiments and samples from patients with HCC. Evidences mentioned above verified that AKT signaling was the cross point in the inhibition mechanism of CREB3 in HCC. Thus, it might play a crucial role in therapy of progenitor subclass of HCC. However, insufficient sample size and lacking of prospective cohort study limited application of our findings to clinical diagnosis and treatment. Further clinical study of CREB3 is needed to confirm its effectiveness in HCC therapy.

INSR was suggested to exist two isoforms, including insulin receptor‐A (INSR‐A) and insulin receptor‐B (INSR‐B), which resulted from alternative splicing of exon 11.^33^ Lacking of exon 11 encoding 12 amino acids located at the carboxy‐terminal domain of extracellular subunits, INSR‐A has high affinity to insulin and IGFs and contributes to fetal development and tumor progression,^34^ while INSR‐B only binds to insulin and regulates glucose and lipid metabolism. Type 2 diabetes mellitus (T2DM) patients with hyperinsulinemia and insulin resistance (IR) are common. Researches have shown that T2DM was suggested to be an independent risk factor of HCC development^35^ and patients with poorly controlled diabetes were more susceptible to HCC.^36^ Meanwhile, INSR‐A/INSR‐B ratio was increased in T2DM patients compared with nondiabetics^37^ and hyperinsulinemia was proved to be correlated with INSR‐A expression,^38^ which indicated hyperinsulinemia promoted exon 11 skipping in INSR. Interestingly, overexpression of INSR‐A and increasing of INSR‐A/INSR‐B ratio were also observed in HCC.^39^ Insulin was suggested to promote tumor progression through INSR‐A with activation of AKT/mTOR signaling.^8^, ^40^ Numerous researches have clearly demonstrated that insulin might promote tumor progression by INSR and not by cross talk with insulin like growth factor 1 receptor (IGF1R).^41^, ^42^ Thus, in the pathological condition of hyperinsulinemia, insulin mainly stimulates HCC progression through interaction with INSR. For therapy of HCC patients with hyperinsulinemia or IR, inhibition of INSR signaling appear to be particularly important. It is worth noting that CREB3 probably suppresses INSR signaling by binding to intracellular domain of INSR‐A and INSR‐B, which provides novel insight for therapy of HCC patients with hyperinsulinemia.

Endogenous ligands of INSR mainly includes insulin, insulin like growth factor 1 (IGF1), IGF2.^43^ The binding of INSR with ligands initiate autophosphorylation and internalization process. Autophosphorylation of INSR recruits and phosphorylates IRS1 to activate PI3K/AKT^10^ and Ras/MAPK signaling.^44^ In addition, Src homology 2‐B (SH2‐B) and protein phosphatases are also recruited and regulate downstream signaling transduction.^45^ In early endosome, INSR is then inactivated and sorted. Subsequently, the majority of INSR are recycled back to the plasma membrane and the rest is degraded in last endosome or imported to the nucleus.^46^ Limitation of our research lies in lacking of specific mechanism investigation of CREB3 and INSR. More evidence were needed to illustrate how CREB3 suppresses the binding of INSR and IRS1 and whether CREB3 plays a role in internalization of INSR.

RBM38 is reported to be a tumor suppressor in HCC development.^47^ The molecular mechanism that RBM38 enhance phosphatase and tensin homolog (PTEN) mRNA stability could result in declined phosphorylation of AKT.^48^ We have identified that CREB3 could suppress phosphorylation of AKT via increasing RBM38 expression in the transcription level. This would be an important explanation of the function of CREB3's N‐terminus in nucleus. Considering INSR signaling regulation, AKT acted as a joint of CREB3 regulation in HCC. Our study highlights the significance of AKT in HCC progress and provides more theoretical support for HCC targeted therapy of AKT inhibitor.

## 4 CONCLUSION

In summary, CREB3 is identified as a suppressor of HCC progression in vitro and in vivo, which attributed to decreasing the phosphorylation of AKT by interfering interaction of INSR with IRS1 and transcriptionally activating RBM38. These findings provide novel insight into the mechanism of inhibitive effects on HCC. Drug discovery based on CREB3 is promising for the treatment of patients with HCC.

## 5 METHODS

### 5.1 Patient samples

Human HCC tissues and adjacent nontumor tissues were collected with prior written and informed consent of patients who were underwent hepatectomy at the Hepatic Surgery Center, Tongji Hospital of Huazhong University of Science and Technology (HUST) (Wuhan, China). This study was approved by the Ethics Committee of Tongji Hospital, HUST (TJH‐202207016) and conducted according to the Declaration of Helsinki Principles.

### 5.2 Cell lines and reagents

The hepatoblastoma cell lines HepG2, HCC cell lines Huh7, Hep3B, HLF, HCC‐LM3 (LM3), MHCC97‐H (97H), PLC/PRF/5, and HEK293T (293T) were obtained from the Hepatic Surgery Center, Tongji Hospital, HUST. Huh7, HLF, LM3, 97H, and 293T were cultured in Dulbecco's modified Eagle medium (DMEM; Hyclone, UT, USA) supplemented with 10% fetal bovine serum (FBS) (Gibco, NY, USA) at 37°C. HepG2, Hep3B, and PLC/PRF/5 were cultured in Eagle's Minimum Essential Medium (Hyclone) supplemented with 10% FBS at 37°C. BFA was purchased from MedChemExpress (NJ, USA).

### 5.3 Plasmid construction and siRNAs synthesis

Short hairpin RNAs (shRNAs) targeting CREB3 were cloned into pLKO.1‐TRC cloning vector (#10878; Addgene, Cambridge, MA, USA) for knockdown of CREB3. A scramble sequence was used as negative control. Coding sequence of CREB3 was synthesized (Tsingke Biotechnology, Beijing, China) and constructed into pLenti‐puro (Addgene; #39481) for stable overexpression of CREB3. Empty pLenti‐puro vector was used as control. All the sequences of shRNAs targeting to corresponding gene were listed in Table S7. pMD2.G (Addgene; #12259) and psPAX2 (Addgene; #12260) were purchased from Addgene and were used for package of lentivirus.

For transient transfection, coding sequence of CREB3 was constructed into pcDNA3.1+ vector (Addgene; #2093). Empty pcDNA3.1+ vector was used as control. INSR and IRS1 plasmids were purchased from WZ Biosciences Inc (Shandong, China). pcDNA3.1+ vector inserted by FLAG‐tagged or HA‐tagged INSR and IRS1 were constructed for transient overexpression. All siRNAs targeting to corresponding gene were purchased from RiboBio (Guangzhou, China). The sequences of siRNAs were listed in Table S7. For luciferase assay, pGL4.17 (E6721) and pRL‐TK (E2241) plasmids were purchased from Promega (WI, USA). Promoter sequence of RBM38 was synthesized (Tsingke Biotechnology) and constructed into pGL4.17. Lipofectamine 3000 transfection reagent (Thermo Fisher Scientific, MA, USA) was used for transient transfection.

### 5.4 Lentivirus package and establishment of stable cell clones

pMD2.G, psPAX2, and constructed plasmid vectors were cotransfected into 293T using the Lipofectamine 3000 transfection reagent to generate lentivirus. Supernatant containing lentivirus was collected 2 days after transfection and filtered with a 0.45 µm filter (PALL, NY, USA). Supernatant was added into cells for virus infection. Then, cells infected by lentivirus particles were screened by 5 µg/mL puromycin (Promoter, Wuhan, China) for 2 weeks after infection. Stably knocked down or overexpressed cells were used for the following experiments.

### 5.5 Cells transfection

pcDNA3.1‐CREB3, pcDNA3.1‐INSR‐FLAG/‐HA, pcDNA3.1‐IRS1‐FLAG/‐HA, INSR siRNA, IRS1 siRNA, and RBM38 siRNA were transfected into cells using Lipofectamine 3000 transfection reagent according to manufacturer's instruction.

### 5.6 Cell proliferation assay

Hep3B/shCREB3 (3 × 10^3^ cells/well), HLF/shCREB3 (2 × 10^3^ cells/well), LM3/CREB3 (3 × 10^3^ cells/well), HLF/CREB3 (2 × 10^3^ cells/well), and their control cells were seeded in 96‐well plate. 10% CCK‐8 (Beyotime Institute of Biotechnology, Shanghai, China) was added into cells and incubated in 37°C for 2 h before detection. Absorbance was measured by an enzyme‐linked immunosorbent assay plate reader (Bio‐Tek Elx 800, VT, USA) at 450 nm.

### 5.7 Colony formation assay

Colony formation assay was performed as described previously.^49^ Briefly, cells (1 × 10^3^ cells/well) were seeded in six‐well plate. Cells were fixed with 4% paraformaldehyde and stained with 0.1% crystal violet at day 14. The number of colonies larger than 100 µm were counted for analysis.

### 5.8 Flow cytometry

Stable cell lines (1 × 10^6^ cells/mL) were seeded in six‐well plate and cultured for 24 h. For cell cycle analysis, HCC cells were fixed in 75% ethanol at 4°C overnight, and then washed with precooled phosphate‐buffered saline (PBS) for three times and were resuspended in 500 µL propidium iodide/RNase Staining Buffer (BD Pharmingen^TM^, CA, USA). After incubated for 15 min at 20°C in the dark, samples were detected by a flow cytometer (NovoCyte 451131110185s; ACEA Biosciences®, CA, USA) and analyzed using FlowJo_v10.8.1 Software (BD Biosciences, NJ, USA). For apoptosis analysis, cells were collected using trypsin solution (Thermo Fisher Scientific) without ethylenediaminetetraacetic acid and were washed with precooled PBS for three times. Annexin V‐FITC/PI Apoptosis Detection Kit (Vazyme Biotech Co., Ltd, Nanjing, China) was used for apoptosis staining. Samples were detected by a flow cytometer and analyzed using FlowJo_v10.8.1 Software.

### 5.9 Transwell assay

A 24‐well transwell plate with 8 µm pore size (Corning, NY, USA) were applied for evaluation of migratory and invasive ability. For migration assay, 5 × 10^4^ cells suspended in 250 µL of DMEM were plated into upper chamber, and 500 µL DMEM with 10% FBS was added into lower chamber. For invasion assay, chamber inserts were precoated with 50 µL mixture of Matrigel (BD Biosciences) and DMEM in a ratio of 1:1 for overnight. 4 × 10^4^ cells were seeded in upper chamber. After 24 h (migration assay) or 72 h (invasion assay), cells were fixed with 4% paraformaldehyde, and stained with 0.1% crystal violet. Then, cells on the top side of insert were scraped off and then cells on the other side were observed under Nikon Digital ECLIPSE C1 system (Nikon Corporation, TKY, Japan). Three random fields in each replicate wells were photographed for statistical analysis. Stained cells were counted using the Image‐Pro Plus v6.0 software (Media Cybernetics Inc, MD, USA).

### 5.10 Tumorigenicity and metastasis assays in vivo

Animal assays were conducted on adherence to Wuhan Medical Experimental Animal Care Guidelines and were approved by the Ethic Committee of Tongji Hospital of HUST. Male BALB/c nude mice were fed under conditions of specific pathogen‐free.

For tumorigenicity assay in vivo, 2 × 10^6^ cells of HCC were suspended in 100 µL DMEM medium without serum and injected into flanks subcutaneous of nude mice. Mice injected with cells were monitored every 3 days and were sacrificed 4 weeks later. Major axis and minor axis were measured, and the volume was calculated: *V* (volume, mm^3^) = 0.5 × *L* (length, mm) × *W* ^2^ (width, mm^2^). After sacrificed, tumors were taken out, weighed, and photographed. Part of tumor tissues were fixed with 4% formalin for 3 days and embedded in paraffin to prepare for IHC staining. Antibodies used for IHC are listed in Table S8.

For metastasis assay in vivo, 1 × 10^6^ HCC cells were suspended in 100 µL DMEM medium and were injected into caudal vein of 4‐week‐old male BALB/C nude mice. After 6 weeks, all mice were sacrificed. Lung of each mouse was separated, fixed with 4% formalin, embedded in paraffin, and sectioned for H&E staining. Metastatic foci of each section were counted for analysis.

### 5.11 mRNA sequencing

Stable overexpressing of CREB3 cells and control cells were lysed in TRIzol reagent (Invitrogen, CA, USA). Extraction of RNA, library construction, high‐throughput sequencing, and data analysis were performed by Biotechnology Corporation (Shanghai, China). DEGs in mRNA levels between control and CREB3‐overexpressing cells were identified using the “edgeR” Bioconductor package. A *p* value cutoff of 0.05 and a fold‐change cutoff of 1.5 were used to evaluate statistical significance of mRNA expression differences. KEGG and GO analysis were performed using “clusterProfiler” Bioconductor package.

### 5.12 Total RNA isolation and qRT‐PCR

Total RNA was extracted by FastPure^®^ Cell/Tissue Total RNA Isolation Kit V2 (Vazyme Biotech Co., Ltd) according to manufacturer's instructions. cDNA was obtained using FastQuant RT kit (Tiangen, Beijing, China). qRT‐PCR was performed using SuperReal PreMix Plus (Tiangen) on ABI ViiA 7 Dx instrument (Applied Biosystems, CA, USA). Relative mRNA expression levels were identified by comparative threshold cycle (2^−ΔΔCT^). Primers were listed in Table S7.

### 5.13 Dual luciferase reporter assay

Dual luciferase reporter assay was performed as described previously.^50^ Luciferase activity was measured in Dual‐Luciferase^®^ Reporter 1000 Assay System (Promega) using GloMax 20/20 Luminometer (Promega) according to manufacturer's instructions. All luciferase activities were normalized to Renilla activity.

### 5.14 Chromatin immunoprecipitation

ChIP was performed according to the instruction of SimpleChIP^®^ Plus Sonication Chromatin IP Kit (Cell Signaling Technology, MA, USA). 4 × 10^6^ cells were collected and fixed with 1% formaldehyde for 10 min at 20°C to crosslink proteins to DNA. After addition of glycine, cells were lysed in 1 mL of sodium dodecyl sulfate buffer, supplemented with protease inhibitors. After incubation for 10 min on ice, samples were sonicated to obtain shear DNA (about 250−1000 bp). Then, sonicated and cross‐linked chromatin extracts were incubated with anti‐CREB3 antibody or rabbit IgG for 4 h at 4°C with rotation. Protein G magnetic beads (Biolinkedin, Shanghai, China) were added into each immunoprecipitation (IP) reaction. After incubation for 2 h at 4°C with rotation, the supernatant was removed by a magnetic separation rack. Chromatins were eluted from the antibody/Protein G magnetic beads with gentle vortexing (1200 rpm) for 30 min at 65°C. Eluted chromatin supernatant was reversed cross‐links by incubation with 6 µL 5 M NaCl and 2 µL Proteinase K for 2 h at 65°C. Then, immunoprecipitated DNA and input were purified and quantified by qRT‐PCR and DNA agarose gel electrophoresis.

### 5.15 Western blotting and co‐IP

Western blotting and co‐IP assays were conducted as described previously.^21^ Protein A/G magnetic beads were purchased from Biolinkedin. Antibodies used in this study were listed in Table S8. HRP goat anti‐rabbit IgG and goat anti‐mouse IgG were obtained from Jackson ImmunoResearch company (PA, USA). Mouse Anti‐Rabbit IgG LCS and Goat Anti‐Mouse IgG LCS (Abbkine, Wuhan, China) were applied in co‐IP blotting to decrease the interference of heavy (∼50 kDa) of the IgG. The optical densities of protein bands were detected using the image J software (National Institute of Mental Health, USA) and were normalized to loading control respectively. Target protein levels were compared across all lanes relative to those in control lanes.

### 5.16 MS analysis

MS analysis of binding proteins obtained from co‐IP assay using anti‐CREB3 antibody or IgG was performed by MONITOR HELIX BioTech Co., Ltd (Shanghai, China). Samples were prepared for three replicates in each group. Proteins overlapping in IgG group and with less than 10 unique peptides detected were excluded. Potential interacting proteins were listed in Table S6.

### 5.17 IF analysis

IF analysis was carried out as described previously.^21^ In brief, cells were plated on coverslips in 12‐well plate. After treatment, cells were fixed in 4% paraformaldehyde for 20 min, then permeabilized in 0.5% TritonX‐100 solution for 10 min, and incubated in indicated primary antibodies overnight at 4°C. For double IF analysis, a mixture of two antibodies were applied. Then, slides were washed three times and incubated in appropriate second antibody for 4 h at 37°C. Nuclei were stained by 4′,6‐diamidino‐2‐phenylindole (DAPI; Sigma–Aldrich, MO, USA) for 5 min. Images were photographed using confocal laser‐scanning microscopy on ZEISS NOL‐LSM 710 (ZEISS, Oberkochen, Germany).

### 5.18 TMAs and IHC

The construction of TMAs and IHC were performed as described previously.^21^ HCC tissues and adjacent nontumor tissues were collected for construction of a TMAs (Shanghai Biochip Co. Ltd, Shanghai, China). TMAs and IHC were stained using corresponding antibodies according to method mentioned previously.^51^ The antibodies used for TMAs and IHC were listed in Table S8. Representative images were photographed using the Leica DFC450C microscope (Leica, Hessen, Germany) and the LAS version 4.12 processing system, or scanned using Pannoramic MIDI (3DHISTECH, Budapest, Hungary). Score of each section was evaluated with the method that multiplying intensity score by percentage score of positive staining cells.^52^

### 5.19 Statistical analysis

Graphpad prism 9 software (La Jolla, CA, USA) was conducted for statistical analysis. The Kaplan–Meier method was applied for the survival analysis. The “survminer” R package was used to calculate the fittest cutoff value and divided patients into low‐ and high‐expression of genes. Pearson's correlation test was applied to assess correlation for continuous variables. Student's *t*‐test was used to analyze continuous variables that obeyed to a normal distribution, while Mann–Whitney *U* test was used to analyze variables that do not obeyed to a normal distribution. Analysis of variance was used to compare two or more variables in several objects. Pearson chi‐square or Fisher's exact test was applied for categorical variables comparison. Results were presented as mean ± standard error of the mean (SEM). A value of *p* < 0.05 was considered as statistical significance, with *p < 0.05, **p < 0.01, and ***p < 0.001.

## AUTHOR CONTRIBUTIONS

*Conception and design*: Yi He, Bixiang Zhang, Zeyang Ding, Han Li, and Zhao Huang. *Acquisition of data*: Yi He, Shenqi Han, Han Li, Zeyu Chen, Yonglong Pan, Ning Cai, Jingyuan Wen, Jianping Zhao, Ganxun Li and Qiumeng Liu. *Analysis and interpretation of data*: Yi He, Wenlong Jia, Zhao Huang, and Yu Wu. *Drafting of the article*: Yi He, Shenqi Han, and Zhao Huang. *Administrative, technical, or material support*: Zeyu Chen, Yonglong Pan, Ning Cai, Jingyuan Wen, Junnan Liang, Qiumeng Liu, and Han Li. *Study supervision and funding acquisition*: Yi He, Zhao Huang, Zeyang Ding and Bixiang Zhang. All authors have read and approved the final manuscript.

## CONFLICT OF INTEREST STATEMENT

The authors declare no conflict of interest.

## ETHICS STATEMENT

The study was approved by the ethics committee of Tongji Hospital of HUST, approval number: TJ‐IRB20210935; the Committee on the Ethics of Animal Experiments of Tongji Hospital, approval number: S‐106‐20‐10‐0P.

## Supporting information

Supporting Information

## Data Availability

The data that support the findings of this study are available from the corresponding author upon reasonable request. The mRNA sequencing data have been deposited to the Science Data Bank (https://www.scidb.cn/en) with the data DOI 10.57760/sciencedb.09254. The MS proteomics data have been deposited to the ProteomeXchange Consortium (https://proteomecentral.proteomexchange.org) via the iProX partner repository^53^, ^54^ with the dataset identifier PXD052604.
