# Supplementary material for: CREB3 suppresses hepatocellular carcinoma progression by depressing AKT signaling through competitively binding with insulin receptor and transcriptionally activating RNA‐binding motif protein 38
Source: MedComm (2020). 2024 Jul 1;5(7):e633. doi: 10.1002/mco2.633 (PMC11215284; doi:10.1002/mco2.633)
Supplement: Supplementary file 1 — Supporting Information [file MCO2-5-e633-s001.docx]

**CREB3 suppresses hepatocellular carcinoma progression by depressing AKT signaling through competitively binding with insulin receptor and transcriptionally activating RNA-binding motif protein 38**

Yi He^1,2,3,4+^, Shenqi Han^1,3,4+^, Han Li^1,3,4+^, Yu Wu^1,3,4+^, Wenlong Jia^1,3,4^, Zeyu Chen^1,3,4^, Yonglong Pan^1,2,3,4^, Ning Cai^1,3,4^, Jingyuan Wen^1,3,4^, Ganxun Li^1,3,4^, Junnan Liang^1,3,4^, Jianping Zhao^1,3,4^, Qiumeng Liu^1,3,4^, Huifang Liang^1,3,4*^, Zeyang Ding^1,3,4*^, Zhao Huang^1,3,4*^, Bixiang Zhang^1,3,4*^

^1^Hepatic Surgery Center, Tongji Hospital, Tongji Medical College, Huazhong University of Science and Technology, 430030, Wuhan, China

^2^Department of Pediatric Surgery, Tongji Hospital, Tongji Medical College, Huazhong University of Science and Technology, 430030, Wuhan, China

^3^Clinical Medical Research Center of Hepatic Surgery at Hubei Province, 430030, Wuhan, China

^4^Hubei Key Laboratory of Hepato-Pancreatic-Biliary Diseases, Tongji Hospital, Tongji Medical College, Huazhong University of Science and Technology, 430030, Wuhan, China

^+^These authors contribute equally

^*^Corresponding authors

**Supplementary figure and figure legends**


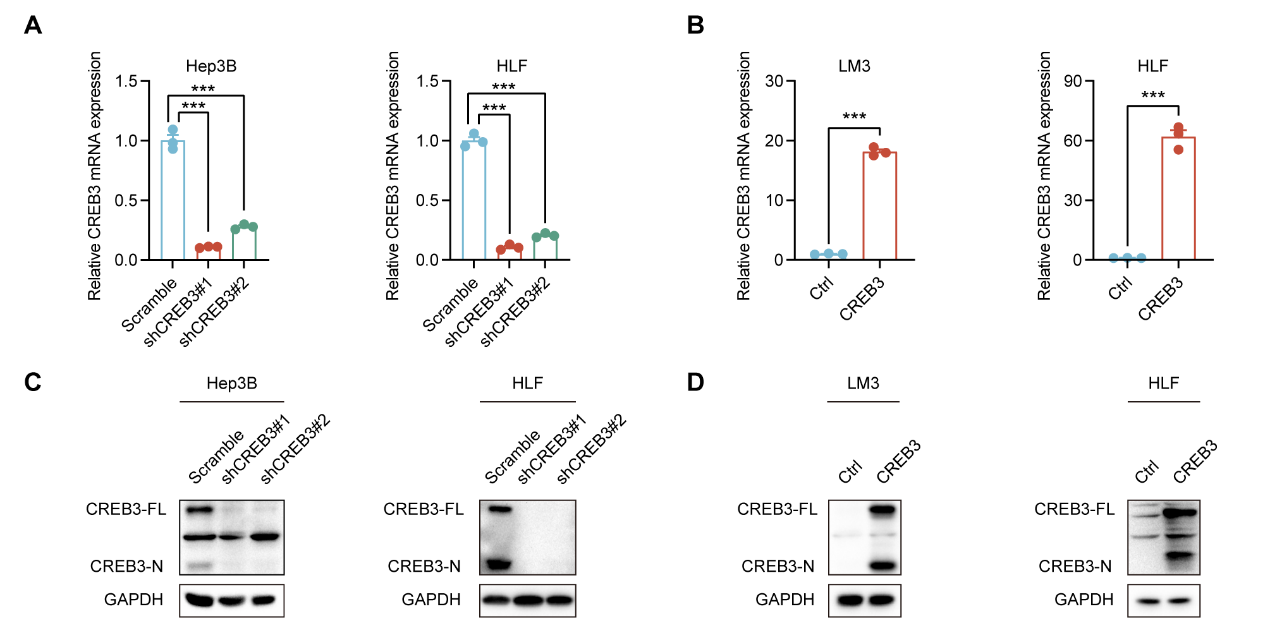


**Fig.S1 Contruction of HCC cell lines with stable overexpression or knockdown of CREB3.** (A, B) qRT-PCR analysis of CREB3 knockdown or overexpression efficiency in indicated HCC cells. Data was shown as the fold change to their respective control cells. (C, D) Western blotting analysis of CREB3 knockdown or overexpression efficiency in indicated HCC cells. GAPDH as loading control. Data was represented as mean ± SEM. ****P* < 0.001.

**
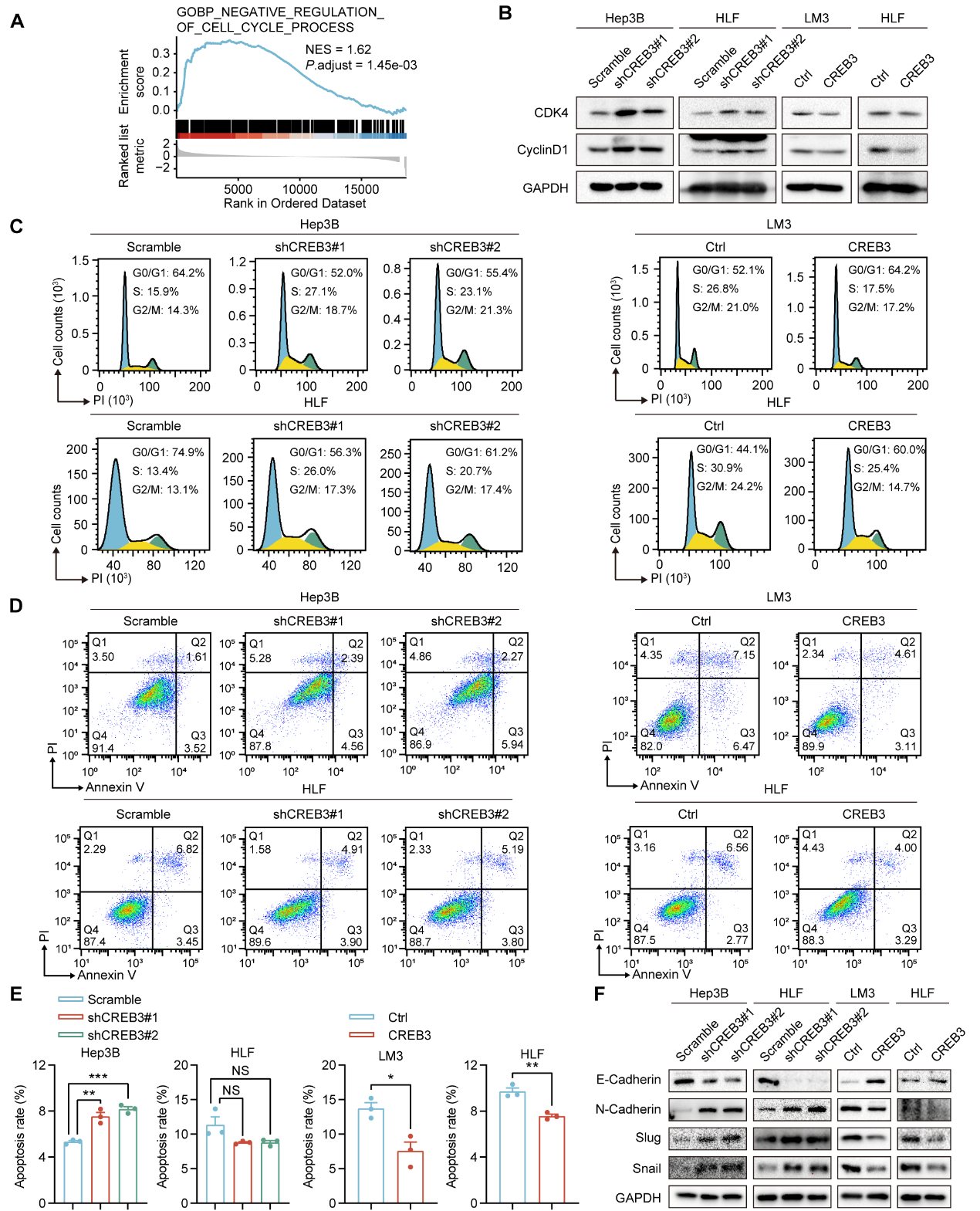
**

**Fig.S2 CREB3 impedes G1 to S phase transition through reducing expression of CDK4 and Cyclin D1.** (A) GSEA analysis for CREB3 using TCGA database in cell cycle process. (B) Western blotting analysis of indicated proteins in HCC cells with CREB3 knockdown or overexpression. (C) Representative flow cytometry assay of cell cycle in indicated cells. (D) Representative apoptosis assay in indicated HCC cells. (E) Statistical analysis of apoptosis in indicated cells with CREB3 knockdown or overexpression (n = 3). (F) Western blotting analysis of EMT related proteins in indicated HCC cells. GAPDH as loading control in (B, F). Data was represented as mean ± SEM. **P* < 0.05, ***P* < 0.01, ****P* < 0.001. NS, no significance. EMT, Epithelial-Mesenchymal transition.


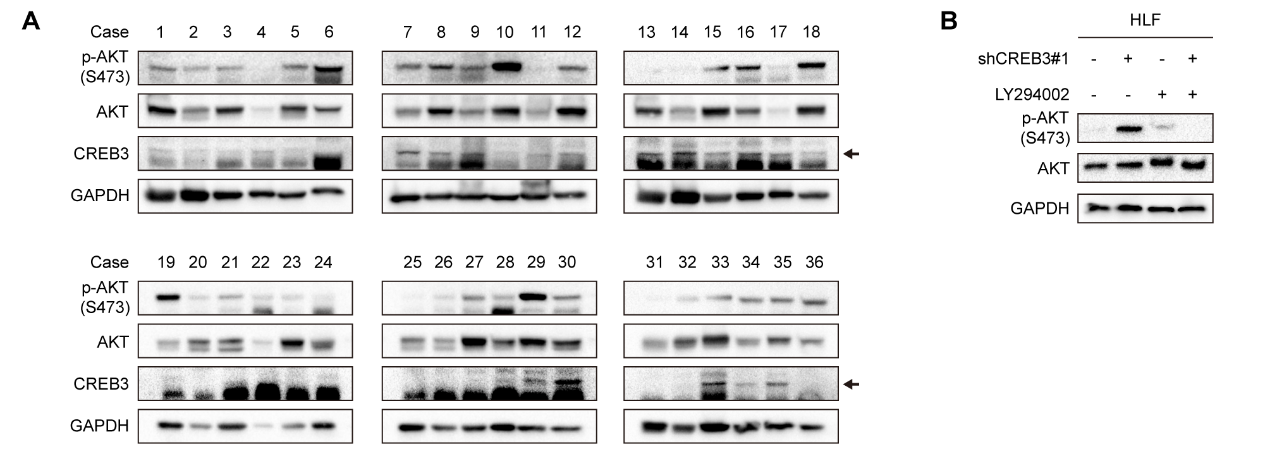


**Fig.S3 CREB3 is negatively correlated with phosphorylation of AKT.** (A) Western blotting analysis of indicated proteins in HCC tissues (n = 36). Band pointed out by arrow indicated CREB3. (B) Western blotting analysis of p-AKT (Ser^473^), total AKT and GAPDH in stable CREB3 knockdown HLF cells with or without LY294002 treatment. GAPDH as loading control.


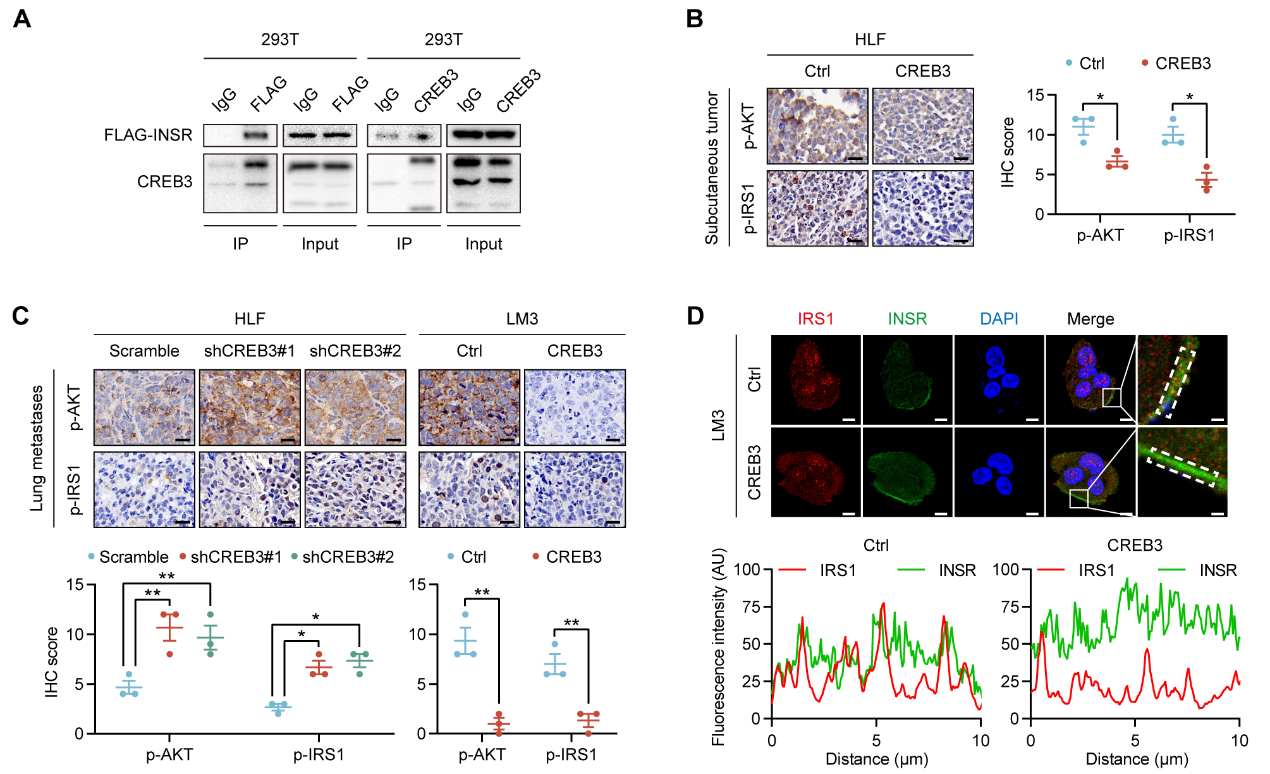


**Fig.S4 CREB3 binds competitively with IRS1 to INSR.** (A) Co-IP analysis of the binding between exogenous FLAG-INSR and CREB3 in co-transfected HEK293T (293T) cells. (B, C) Representative images and quantification of p-AKT (Ser^473^) and p-IRS1 (Ser^307^) staining of subcutaneous tumor (B) and lung metastases (C) in the indicated groups (n = 3). Scale bar: 20 μm. (D) Representative confocal images and line scans analysis of IRS1 (red) and INSR (green) staining in LM3 cells with CREB3 overexpression or control. Nuclei were counterstained with DAPI (blue). Scale bar: 10 μm, 2 μm. Data was represented as mean ± SEM. **P* < 0.05, ***P* < 0.01.

**
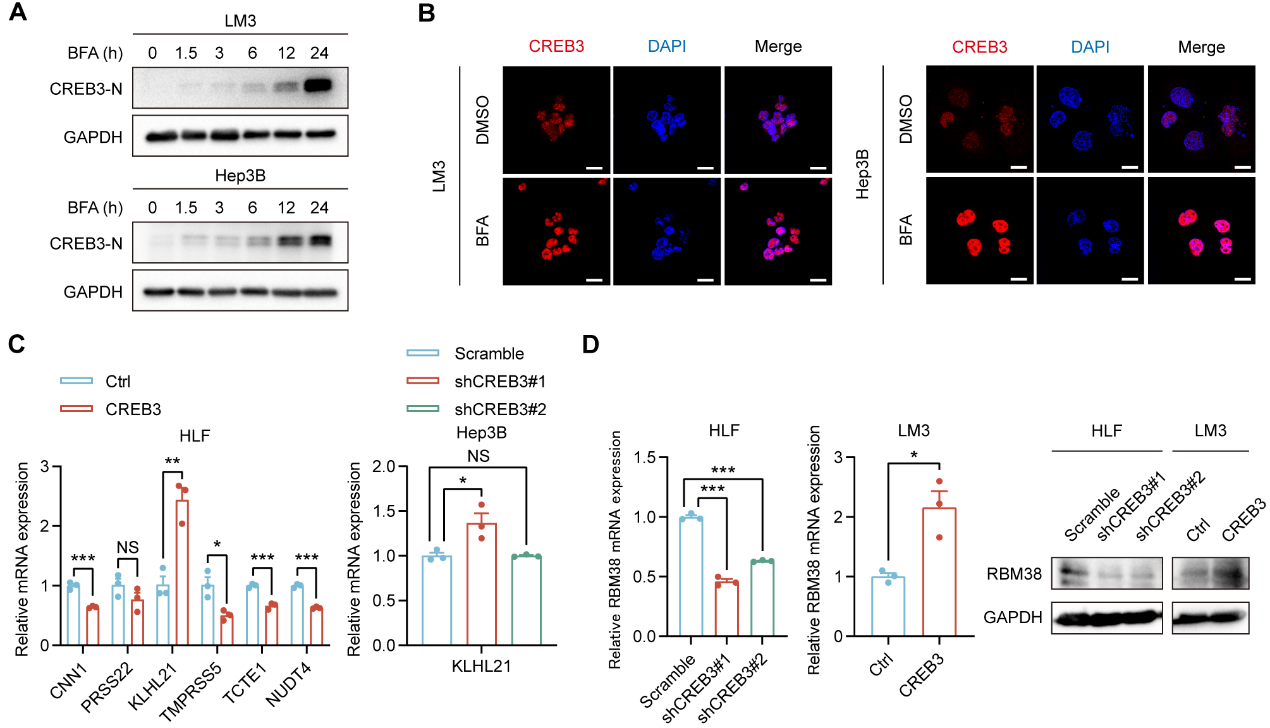
**

**Fig.S5 N-terminus of CREB3 is imported to nucleus with treatment of BFA.** (A) Western blotting detection of N-terminus of CREB3 (CREB3-N) with treatment of BFA at indicated time. (B) Immunofluorescent analysis of LM3 and Hep3B treated with BFA and stained by CREB3 antibody (Red). Nuclei were counterstained with DAPI (blue). Scale bar: 20 μm. (C) The mRNA expression of indicated candidate genes in HCC cells with CREB3 knockdown or overexpression. (D) qRT-PCR and western blotting analysis of RBM38 in indicated HCC cells with CREB3 knockdown or overexpression. GAPDH as loading control in (A, D). Data was represented as mean ± SEM. **P* < 0.05, ***P* < 0.01, ****P* < 0.001. NS, no significance.


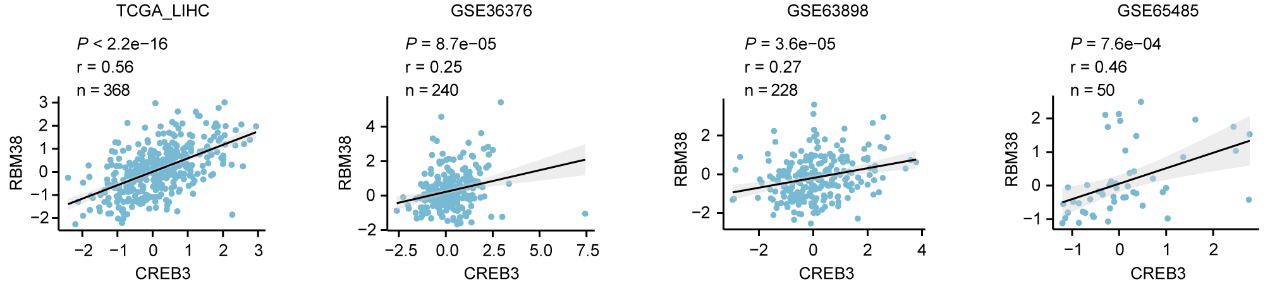


**Fig.S6 The expression of CREB3 is positively correlated with RBM38.** (A) Scatterplots showing the positive correlation of CREB3 and RBM38 expression in TCGA-LIHC and GEO cohorts. Statistical analysis was performed using Pearson’s correlation.


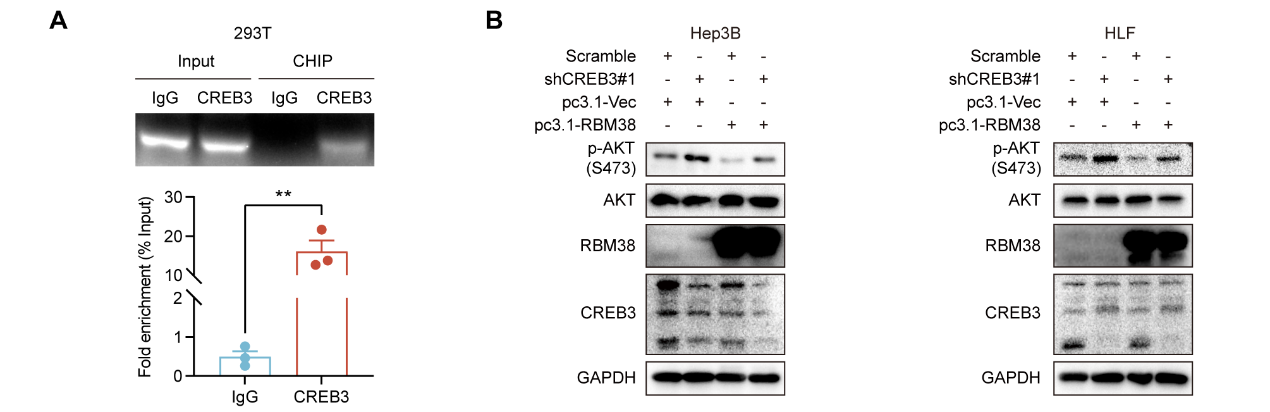


**Fig.S7 CREB3 suppresses AKT phosphorylation by transactivating RBM38.** (A) Exogenous ChIP experiment of CREB3 binding to RBM38 promoter in 293T cells. qRT-PCR detection and agarose gel electrophoresis of PCR fragments after ChIP. (B) Western blotting analysis of indicated protein in CREB3 stably knocked down or negative control Hep3B and HLF cells transfected with pcDNA3.1-RBM38 (pc3.1-RBM38) or pcDNA3.1-vector plasmid (pc3.1-Vec) for 48 h. GAPDH as loading control. Data was represented as mean ± SEM. ***P* < 0.01.


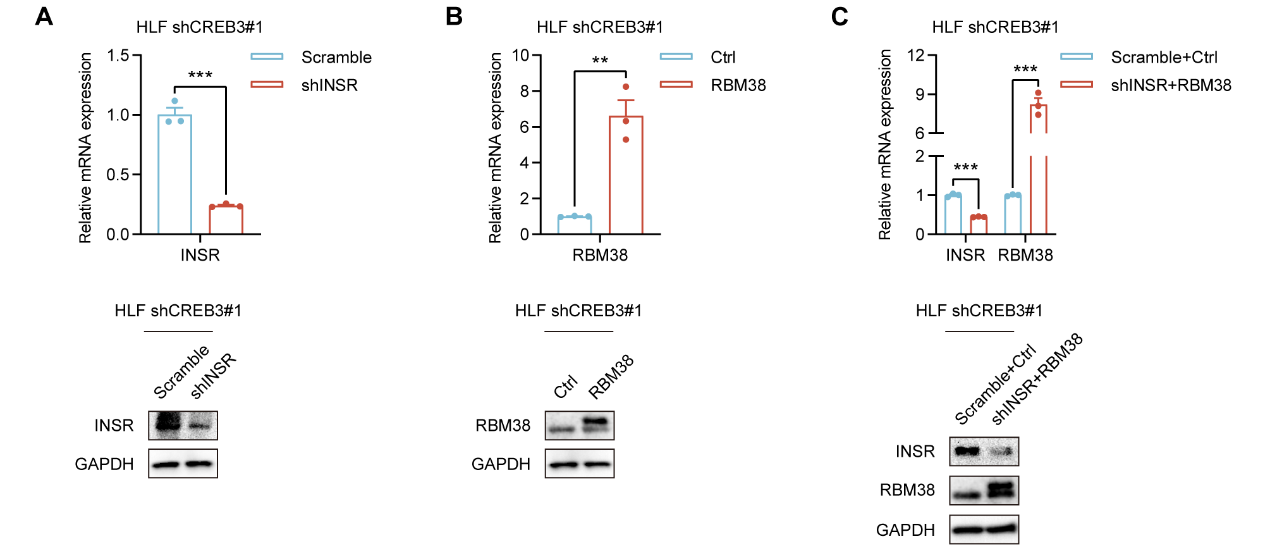


**Fig.S8 Knockdown of INSR or overexpression of RBM38 independently rescues effects induced by CREB3 knockdown.** (A-C) qRT-PCR and western blotting detection of HLF with CREB3 stably knocked down with or without INSR knockdown or/and RBM38 overexpression. GAPDH as loading control. Data was represented as mean ± SEM. ***P* < 0.01, ****P* < 0.001.

**Supplementary Table 1. Clinicopathologic characteristics of patients with hepatocellular carcinoma in cohort 1 (n= 109).**

| **Clinicopathological  variables** | **Number (n=109)** | **Percentage** |  |
| --- | --- | --- | --- |
|  |  |  |  |
| **Gender** |  |  |  |
| Male | 89 | 81.7% |  |
| Female | 20 | 18.3% |  |
| **Age** |  |  |  |
| ≤ 50 | 54 | 49.5% |  |
| > 50 | 55 | 50.5% |  |
| **AFP (μg/L)** |  |  |  |
| ≤ 20 | 28 | 25.7% |  |
| > 20 | 81 | 74.3% |  |
| **ALT (U/L)** |  |  |  |
| ≤ 41 | 80 | 73.4% |  |
| > 41 | 29 | 26.6% |  |
| **AST (U/L)** |  |  |  |
| ≤ 40 | 76 | 69.7% |  |
| > 40 | 33 | 30.3% |  |
| **ALP (U/L)** |  |  |  |
| ≤ 130 | 93 | 85.3% |  |
| > 130 | 16 | 14.7% |  |
| **HBV** |  |  |  |
| Negative | 0 | 0.0% |  |
| Positive | 109 | 100.0% |  |
| **HCV** |  |  |  |
| Negative | 109 | 100.0% |  |
| Positive | 0 | 0.0% |  |
| **Cirrhosis** |  |  |  |
| No | 30 | 27.5% |  |
| Yes | 79 | 72.5% |  |
| **Tumor size (cm)** |  |  |  |
| ≤ 5 | 39 | 35.8% |  |
| > 5 | 70 | 64.2% |  |
| **Tumor number** |  |  |  |
| Single | 85 | 78.0% |  |
| Multiple | 24 | 22.0% |  |
| **Tumor encapsulation** |  |  |  |
| No | 49 | 45.0% |  |
| Yes | 60 | 55.0% |  |
| **Vascular invasion** |  |  |  |
| No | 86 | 78.9% |  |
| Yes | 23 | 21.1% |  |
| **Child-Pugh** |  |  |  |
| A | 105 | 96.3% |  |
| B | 4 | 3.7% |  |
| **Differentiation** |  |  |  |
| Ⅰ+Ⅱ | 77 | 70.6% |  |
| Ⅲ+Ⅵ | 32 | 29.4% |  |
| **BCLC stage** |  |  |  |
| 0+A | 71 | 65.1% |  |
| B+C | 38 | 34.9% |  |
| **TNM stage** |  |  |  |
| Ⅰ+Ⅱ | 80 | 73.4% |  |
| Ⅲ+Ⅵ | 29 | 26.6% |  |

**Supplementary Table 2. Correlation between relative CREB3 expression and clinicopathologic characteristics of patients with hepatocellular carcinoma in cohort 1 (n= 109).**

| **Clinicopathological**  **variables** | **Relative CREB3 expression** | | ***P* value** |
| --- | --- | --- | --- |
|  | **Low (n=61)** | **High (n=48)** |  |
| **Gender** |  |  |  |
| Male | 46 | 43 | 0.099 |
| Female | 15 | 5 |  |
| **Age** |  |  |  |
| ≤ 50 | 36 | 18 | **0.042** |
| > 50 | 25 | 30 |  |
| **AFP (μg/L)** |  |  |  |
| ≤ 20 | 7 | 21 | **< 0.001** |
| > 20 | 54 | 27 |  |
| **ALT (U/L)** |  |  |  |
| ≤ 41 | 46 | 34 | 0.750 |
| > 41 | 15 | 14 |  |
| **AST (U/L)** |  |  |  |
| ≤ 40 | 40 | 37 | 0.258 |
| > 40 | 21 | 12 |  |
| **ALP (U/L)** |  |  |  |
| ≤ 130 | 54 | 39 | 0.428 |
| > 130 | 7 | 9 |  |
| **HBV** |  |  |  |
| Negative | 0 | 0 | - |
| Positive | 61 | 48 |  |
| **HCV** |  |  |  |
| Negative | 61 | 48 | - |
| Positive | 0 | 0 |  |
| **Cirrhosis** |  |  |  |
| No | 18 | 12 | 0.759 |
| Yes | 43 | 36 |  |
| **Tumor size** |  |  |  |
| ≤ 5 | 16 | 23 | **0.032** |
| > 5 | 45 | 25 |  |
| **Tumor number** |  |  |  |
| Single | 46 | 39 | 0.619 |
| Multiple | 15 | 9 |  |
| **Tumor encapsulation** |  |  |  |
| No | 38 | 11 | **< 0.001** |
| Yes | 23 | 37 |  |
| **Vascular invasion** |  |  |  |
| No | 49 | 46 | **0.035** |
| Yes | 12 | 2 |  |
| **Child-Pugh** |  |  |  |
| A | 59 | 46 | 1.000 |
| B | 2 | 2 |  |
| **Differentiation** |  |  |  |
| Ⅰ+Ⅱ | 36 | 41 | **0.005** |
| Ⅲ+Ⅵ | 25 | 7 |  |
| **BCLC stage** |  |  |  |
| 0+A | 34 | 37 | **0.034** |
| B+C | 27 | 11 |  |
| **TNM stage** |  |  |  |
| Ⅰ+Ⅱ | 41 | 39 | 0.153 |
| Ⅲ+Ⅵ | 20 | 9 |  |

**Supplementary Table 3. Univariate and multivariate analysis of factors associated with survival and recurrence of patients with hepatocellular carcinoma in cohort 1 (n= 109).**

|  | **Survival** | | | | | |
| --- | --- | --- | --- | --- | --- | --- |
|  | **Univariate analysis** | | | **Multivariate analysis** | | |
|  | **HR** | **95% CI** | **P value** | **HR** | **95% CI** | ***P* value** |
| **Gender (male vs female）** |  |  | 0.588 |  |  |  |
| **Age (> 50 vs ≤ 50)** |  |  | 0.460 |  |  |  |
| **AFP (> 20 vs ≤ 20 μg/L)** |  |  | 0.281 |  |  |  |
| **ALT (> 41 vs ≤ 41 U/L)** |  |  | 0.826 |  |  |  |
| **ALP (> 130 vs ≤ 130 U/L)** |  |  | 0.271 |  |  |  |
| **Cirrhosis (yes vs no)** |  |  | 0.599 |  |  |  |
| **Tumor size (> 5 vs ≤ 5 cm)** | 3.319 | 1.380-7.980 | **0.007** |  |  | 0.107 |
| **Tumor number (multiple vs single)** |  |  | 0.804 |  |  |  |
| **Tumor encapsulation (no vs yes)** | 1.954 | 1.009-3.785 | **0.047** |  |  | 0.974 |
| **Vascular invasion (yes vs no)** | 4.409 | 2.197-8.850 | **< 0.001** | 2.898 | 0.998-8.411 | **0.050** |
| **Differentiation (Ⅲ+Ⅵ vs Ⅰ+Ⅱ)** | 1.469 | 0.743-2.907 | 0.269 |  |  |  |
| **Child-Pugh (B vs A)** | 3.538 | 1.064-11.771 | **0.039** |  |  | 0.154 |
| **BCLC stage (B+C vs 0+A)** | 2.770 | 1.434-5.350 | **0.002** |  |  | 0.772 |
| **TNM stage (Ⅲ+Ⅵ vs Ⅰ+Ⅱ)** | 2.702 | 1.396-5.227 | **0.003** |  |  | 0.773 |
| **CREB3 expression in tumor (high vs low)** | 0.314 | 0.147-0.670 | **0.003** | 0.437 | 0.193-0.988 | **0.047** |

|  | **Recurrence** | | | | | |
| --- | --- | --- | --- | --- | --- | --- |
|  | **Univariate analysis** | | | **Multivariate analysis** | | |
|  | **HR** | **95% CI** | **P value** | **HR** | **95% CI** | ***P* value** |
| **Gender (male vs female）** | 1.122 | 0.549-2.290 | 0.752 |  |  |  |
| **Age (> 50 vs ≤ 50)** | 0.879 | 0.520-1.486 | 0.631 |  |  |  |
| **AFP (> 20 vs ≤ 20 μg/L)** | 1.249 | 0.671-2.323 | 0.483 |  |  |  |
| **ALT (> 41 vs ≤ 41 U/L)** | 0.876 | 0.478-1.608 | 0.670 |  |  |  |
| **ALP (> 130 vs ≤ 130 U/L)** | 1.365 | 0.689-2.705 | 0.373 |  |  |  |
| **Cirrhosis (yes vs no)** | 1.225 | 0.669-2.245 | 0.510 |  |  |  |
| **Tumor size (> 5 vs ≤ 5 cm)** | 3.634 | 1.828-7.223 | **< 0.001** | 2.643 | 1.241-5.623 | **0.012** |
| **Tumor number (multiple vs single)** | 1.500 | 0.829-2.712 | 0.180 |  |  |  |
| **Tumor encapsulation (no vs yes)** | 2.213 | 1.303-3.758 | **0.003** |  |  | 0.575 |
| **Vascular invasion (yes vs no)** | 2.779 | 1.561-4.945 | **0.001** |  |  | 0.330 |
| **Differentiation (Ⅲ+Ⅵ vs Ⅰ+Ⅱ)** | 1.446 | 0.829-2.522 | 0.193 |  |  |  |
| **Child-Pugh (B vs A)** | 3.873 | 1.376-10.895 | **0.010** | 3.973 | 1.280-12.335 | **0.017** |
| **BCLC stage (B+C vs 0+A)** | 2.504 | 1.477-4.243 | **0.001** |  |  | 0.967 |
| **TNM stage (Ⅲ+Ⅵ vs Ⅰ+Ⅱ)** | 2.652 | 1.554-4.527 | **< 0.001** |  |  | 0.738 |
| **CREB3 expression in tumor (high vs low)** | 0.384 | 0.218-0.676 | **0.001** | 0.447 | 0.237-0.841 | **0.013** |

**Supplementary Table 4. List of top 30 enriched GO pathways in RNA-seq of HLF cells with CREB3 overexpression.**

| **GO pathway** | **Rich Factor** | ***P* value (-log 10)** |
| --- | --- | --- |
| Regulation of protein kinase B signaling | 4.781849 | 3.115715 |
| Maintenance of location in cell | 4.705947 | 2.728804 |
| Maintenance of protein location in cell | 4.205314 | 2.178894 |
| Steroid binding | 4.075253 | 2.12339 |
| Alcohol binding | 4.075253 | 2.12339 |
| Negative regulation of inflammatory response | 3.913857 | 2.052394 |
| Protein kinase B signaling | 3.800957 | 2.556885 |
| Chloride transport | 3.561257 | 1.888397 |
| Drug binding | 3.437387 | 1.827628 |
| Motor activity | 3.43142 | 2.070915 |
| Regulation of stem cell differentiation | 3.213817 | 1.713371 |
| Heparin binding | 3.012954 | 1.812083 |
| ATP metabolic process | 2.882392 | 2.110467 |
| Inorganic anion transport | 2.823568 | 1.49822 |
| Regulation of muscle contraction | 2.745136 | 1.452311 |
| Serine-type endopeptidase activity | 2.729969 | 1.621558 |
| Negative regulation of MAPK cascade | 2.670943 | 1.407992 |
| Neuron projection extension | 2.670943 | 1.407992 |
| Regulation of muscle system process | 2.573565 | 1.510226 |
| Purine nucleoside metabolic process | 2.498382 | 2.061982 |
| Nucleoside metabolic process | 2.43411 | 2.129139 |
| Serine-type peptidase activity | 2.398662 | 1.380207 |
| Microtubule | 2.112646 | 1.592054 |
| Positive regulation of cellular component Biogenesis | 2.08296 | 1.554183 |
| Neuronal cell body | 2.063629 | 1.529391 |
| Ribonucleotide metabolic process | 2.016834 | 1.662579 |
| Purine nucleotide metabolic process | 1.994631 | 1.628748 |
| Ribose phosphate metabolic process | 1.972911 | 1.595518 |
| Cell body | 1.972553 | 1.50339 |
| Endopeptidase activity | 1.946223 | 1.376633 |

**Supplementary Table 5. List of top 30 enriched KEGG pathways in RNA-seq of HLF cells with CREB3 overexpression.**

| **KEGG pathway** | **Rich Factor** | ***P* value**  **(-log 10)** |
| --- | --- | --- |
| Glycosaminoglycan biosynthesis-chondroitin sulfate/ Dermatan sulfate | 12.8037 | 2.903148 |
| Type II diabetes mellitus | 5.334877 | 1.837615 |
| Aldosterone synthesis and secretion | 4.742112 | 2.03246 |
| Viral myocarditis | 4.41507 | 1.617592 |
| Hepatitis B | 4.38483 | 2.521116 |
| p53 signaling pathway | 3.711218 | 1.420345 |
| Hippo signaling pathway | 3.325637 | 1.73158 |
| Complement and coagulation cascades | 3.241444 | 1.27023 |
| Cell cycle | 3.09767 | 1.414765 |
| AMPK signaling pathway | 3.09767 | 1.414765 |
| Hypertrophic cardiomyopathy (HCM) | 3.08523 | 1.216321 |
| Taste transduction | 3.08523 | 1.216321 |
| TGF-beta signaling pathway | 3.048501 | 1.203325 |
| Insulin secretion | 3.012636 | 1.190512 |
| Small cell lung cancer | 2.977606 | 1.177878 |
| Dilated cardiomyopathy | 2.877237 | 1.141007 |
| Estrogen signaling pathway | 2.586607 | 1.028184 |
| Melanogenesis | 2.560741 | 1.017673 |
| Glucagon signaling pathway | 2.535387 | 1.00729 |
| Proteoglycans in cancer | 2.522897 | 1.27617 |
| MAPK signaling pathway | 2.51053 | 1.404921 |
| Insulin resistance | 2.349303 | 0.928569 |
| TNF signaling pathway | 2.327946 | 0.919237 |
| Cholinergic synapse | 2.306974 | 0.910012 |
| Leukocyte transendothelial migration | 2.170119 | 0.848252 |
| Huntington,s disease | 1.990213 | 0.837112 |
| HTLV-I infection | 1.98507 | 0.914476 |
| Pathways in cancer | 1.935069 | 1.028605 |
| cAMP signaling pathway | 1.930207 | 0.800234 |
| PI3K-Akt signaling pathway | 1.882898 | 0.909699 |

**Supplementary Table 6. Shortlisted candidate proteins from co-IP mass spectrometry using CREB3 as bait (the count of unique peptides ≥ 10)**

| **Gene Symbol** | **Aliase** | **Gene ID** | **Function** |
| --- | --- | --- | --- |
| KRT1 | keratin, type II cytoskeletal 1 | 3848 | carbohydrate binding, protein binding |
| KRT10 | keratin, type I cytoskeletal 10 | 3858 | structural molecule activity, structural constituent of skin epidermis |
| KRT2 | keratin, type II cytoskeletal 2 epidermal | 3849 | structural molecule activity, structural constituent of cytoskeleton |
| HCFC1 | host cell factor 1 | 3054 | chromatin binding, DNA-binding transcription factor activity |
| ALB | albumin | 213 | enzyme binding, protein-folding chaperone binding |
| INSR | insulin receptor | 3643 | protein tyrosine kinase activity, transferring phosphorus-containing groups, transferase activity |
| HNRPU | heterogeneous nuclear ribonucleoprotein U | 3192 | RNA binding, kinase activity |

**Supplementary Table 7. Gene specific primers used for qRT-PCR and sequences of siRNA and shRNA.**

| **Gene name** |  | **Oligo sequence** |
| --- | --- | --- |
| CREB3 | Forward | 5’- ATGGAGCTGGAATTGGATGCT -3’ |
|  | Reverse | 5’- TACTTCCCAGTCGCTCGGT -3’ |
| IRS1 | Forward | 5’- GCGTGTCAGTTTGTCAAATACCCCA -3’ |
|  | Reverse | 5’- GAGGTCATTTAGGTCTTCATTCTGC -3’ |
| INSR | Forward | 5’- ACAGACTATTTAGACGTCCCGT -3’ |
|  | Reverse | 5’- CCATCTGGCTGCCTCTTTCT -3’ |
| RBM38 | Forward | 5’- GATCCAGCGGACTTACGGGC -3’ |
|  | Reverse | 5’- GGGTAGCTGTAGCCCACGAA -3’ |
| GAPDH | Forward | 5’- CTGGGCTACACTGAGCACC -3’ |
|  | Reverse | 5’- AAGTGGTCGTTGAGGGCAATG -3’ |
| Scramble | Top | 5’- CCGGCCAGACCACTACTGAATATAACTCG  AGTTATATTCAGTAGTGGTCTGGTTTTT -3’ |
|  | Bottom | 5’- AATTAAAAACCAGACCACTACTGAATATA  ACTCGAGTTATATTCAGTAGTGGTCTGG -3’ |
| shCREB3#1 | Top | 5’- CCGGCGGAGGAAGATTCGAAATAAACTCG  AGTTTATTTCGAATCTTCCTCCGTTTTT -3’ |
|  | Bottom | 5’- AATTAAAAACGGAGGAAGATTCGAAATAA  ACTCGAGTTTATTTCGAATCTTCCTCCG -3’ |
| shCREB3#2 | Top | 5’- CCGGACAGATACTCAGGCTAGATATCTCG  AGATATCTAGCCTGAGTATCTGTTTTTT -3’ |
|  | Bottom | 5’- AATTAAAAAACAGATACTCAGGCTAGATA  TCTCGAGATATCTAGCCTGAGTATCTGT -3’ |
| NC siRNA | Sense | 5’- UUCUCCGAACGUGUCACGUTT -3’ |
|  | Antisense | 5’- ACGUGACACGUUCGGAGAATT -3’ |
| siIRS1 | Sense | 5’- GGGCUGACUCCAAGAACAATT -3’ |
|  | Antisense | 5’- UUGUUCUUGGAGUCAGCCCTT -3’ |
| siINSR | Sense | 5’- CUACGUGACAGACUAUUUA -3’ |
|  | Antisense | 5’- UAAAUAGUCUGUCACGUAG -3’ |

**Supplementary Table 8. Antibodies used in this study.**

| **Antigens** | **Lot, manufacturers** | **Application** |
| --- | --- | --- |
| CREB3 | 11275-1-AP, proteintech | 1:1000 for WB, 1:200 for IHC, 1: 50 for IF, 1:200 for IP |
| p-AKT (Ser^473^) | 4060, CST | 1:1000 for WB |
| AKT | 4691, CST | 1:1000 for WB |
| INSR | 20433-1-AP, Proteintech | 1:1000 for WB |
| INSR | 3025, CST | 1:1000 for WB,1:40 for IP |
| INSR | AHR0271, Invitrogen | 1:20 for IF |
| IRS1 | A16902, Abclonal | 1:1000 for WB,1:100 for IF |
| IRS1 | 3407, CST | 1:1000 for WB |
| p-IRS1 (Ser^307^) | 2381, CST | 1:1000 for WB |
| RBM38 | sc-365898, Santa Cruz | 1:500 for WB, 1:100 for IHC |
| Ki67 | 9027, CST | 1:400 for IHC |
| FLAG | F1804, Sigma-Aldrich | 1:500 for WB, 1:100 for IP |
| HA | H6908, Sigma-Aldrich | 1:1000 for WB,1:100 for IP |
| GAPDH | 60004-1-Ig, Proteintech | 1:10000 for WB |
| CDK4 | 12790, CST | 1:1000 for WB |
| Cyclin D1 | 2978, CST | 1:1000 for WB |
| p-Mek | sc-271914, Santa Cruz | 1:500 for WB |
| Mek | sc-449, Santa Cruz | 1:500 for WB |
| p-ERK | 4370, CST | 1:1000 for WB |
| ERK | 4695, CST | 1:1000 for WB |
| HRP goat anti-rabbit IgG | 111-035-003, Jackson ImmunoResearch | 1:10000 for WB |
| HRP goat anti-mouse IgG | 115-035-003, Jackson ImmunoResearch | 1:10000 for WB |
| Mouse Anti-Rabbit IgG LCS | A25022, Abbkine | 1:2000 for IP |
| Goat Anti-Mouse IgG LCS | A25012, Abbkine | 1:2000 for IP |
| Alexa Fluor 488-conjugated anti-mouse IgG | A-11001, Thermo Fisher | 1:200 for IF |
| Alexa Fluor 594-conjugated anti-rabbit IgG | A-21207, Thermo Fisher | 1:200 for IF |
